# Pyrene-4,5-dione as a Visible-Light Organic Photocatalyst for Photooxidation, Photoredox, Energy Transfer, and Hydrogen Atom Transfer Reactions

**DOI:** 10.1021/acsorginorgau.5c00111

**Published:** 2026-01-06

**Authors:** Rodolfo I. Teixeira, Joseph P. Anslow, Nanci C. de Lucas

**Affiliations:** † Department of Chemical Engineering, 5156 Loughborough University, Loughborough LE11 3TU, U.K.; ‡ Instituto de Química, 28125 Universidade Federal do Rio de Janeiro, Cidade Universitária, Rio de Janeiro, RJ 21941-909, Brazil

**Keywords:** photocatalysis, pyrene-4, 5-dione, energy transfer, electron transfer, HAT catalysis, photooxidation

## Abstract

Pyrene-4,5-dione (PQ) is demonstrated to be a metal-free, visible-light organic photocatalyst capable of mediating four major photochemical activation modes: photooxidation, energy transfer, photoredox, and hydrogen atom transfer (HAT). Under mild conditions using blue LEDs, PQ enables diverse synthetic transformations, including singlet oxygen oxidations, [2 + 2] cycloadditions, C–H fluorination, reductive C–C bond formation, oxidative hydroxylation of boronic acids, and HAT-driven alkylation. Its performance is comparable to benchmark systems, while offering the advantages of visible-light absorption and a greener profile compared to typical metal-complex photocatalysts. PQ can be recovered chromatographically (typically 85–90% mass recovery) with no evidence of structural change or degradation. Further, as a proof-of-concept, the reductive C–C coupling was performed in continuous flow, delivering up to 85% yield at 0.17 mmol h^–1^ in a 4 mL reactor and 83% yield at 0.41 mmol h^–1^ upon numbering-up to a 10 mL reactor. This work highlights the potential of PQ as a practical and versatile photocatalyst for visible-light-driven organic synthesis.

## Graphical abstract

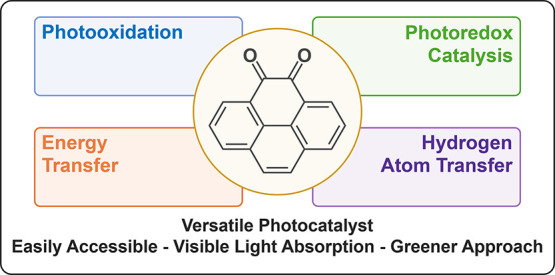

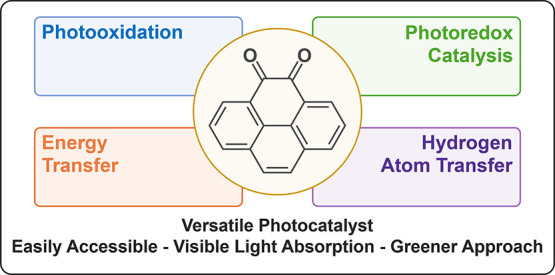

## Introduction

Light-driven catalysis has emerged as a greener and safer alternative to conventional chemical transformations.^1^ Photochemical reactions often exhibit vastly improved selectivity compared to classical thermal methods. This arises from precise control of excitation via wavelength tuning, access to unique excited-state reaction pathways, and operation under mild conditions that suppress side processes.^1–3^ In addition, the use of light enables sustainable process development, especially when combined with continuous flow and highly efficient LEDs.^4^ These advantages have contributed to the widespread adoption of visible-light photocatalysis in both academic and industrial settings.^5–7^

Reactions enabled by visible light and a photocatalyst typically fall into one of four mechanistic classes: (i) singlet oxygen photooxidation, (ii) photoredox, (iii) energy transfer (EnT) or (iv) hydrogen atom transfer (HAT) reactions.^8–12^

Greener and sustainable synthesis of fine chemicals is paramount for a sustainable chemical industry.^13,14^ However, the sustainability of photocatalytic processes is often compromised by low process mass intensity (PMI), particularly due to solvent-intensive purification steps and the reliance on precious-metal-based photocatalysts.^13^ Organic photocatalysts therefore represent a more sustainable alternative, offering lower toxicity and reduced environmental impact.^15–18^

Quinones are especially attractive candidates for organic photocatalysis. Both natural and synthetic quinones exhibit rich photophysical and photochemical behavior.^19–22^ Upon photoexcitation, they undergo efficient intersystem crossing to their triplet excited states^23^ enabling diverse reactivity, including energy transfer (EnT), single electron transfer (SET), proton-coupled electron transfer (PCET), and hydrogen atom abstraction (HAT) with a wide range of substrates. These properties have generated growing interest in quinones as metal-free photocatalysts for synthetic applications.

Among quinone derivatives, anthraquinones and benzoquinones have been the most extensively studied, particularly for their ability to mediate HAT-driven C–H functionalization and photooxidation processes.^12,24–26^ Naphthoquinones have also shown promise as versatile organic photocatalysts.^27,28^ More recently, conjugated polyaromatic quinones have attracted interest for their extended absorption into the visible range and enhanced photoredox performance. For example, 9,10-phenanthrenedione has been used in photoredox, HAT, and photooxidation reactions,^29,30^ While pyrene-1,6-dione and pyrene iminoquinone have been reported as catalysts for aerobic alkylation of C­(sp^3^)–H bonds^31^ and oxidative dehydrogenation of saturated *N*-heterocycles,^32^ respectively.

Pyrene-4,5-dione (PQ) is a particularly promising candidate for visible-light photocatalysis. It exhibits strong absorption in the visible region (λ_max_ ≈ 450 nm), comparable to that of commonly used ruthenium complexes. Its triplet energy (∼2.0 eV)^33^ is similar to that of 9,10-anthraquinone (2.3 eV), and notably higher than that of functionalized anthraquinones such as 1,8-dihydroxyanthraquinone (0.8 eV) and 1-aminoanthraquinone (1.3 eV).^26^ PQ also exhibits a high singlet oxygen quantum yield (Φ_Δ_ = 0.8),^34^ making it a well-suited photosensitizer for ^1^O_2_-mediated photooxidation reactions. Additionally, its excited-state reduction potential (+2.02 V vs SCE) enables oxidative electron transfer with substrates including amines, alcohols, olefins, and arenes.^17^

Although its photochemical properties have been investigated both experimentally and theoretically,^33–36^ its potential in synthetic chemistry remains largely unexplored, having only recently been used as a photocatalyst in cofactor-inspired catalysts exploring its PCET process with benzylic oxidation of alcohols,^37–40^ and the ability of *o*-quinones to form stable dihydroxyl pyrene species via photoreduction.^34^

Despite promising photophysical properties, the synthetic utility of PQ remains largely underexplored. Herein, we report the application of pyrene-4,5-dione as a metal-free photocatalyst across all four major mechanistic classes of visible-light-induced transformations: Photooxidation, Energy transfer (EnT), Photoredox, and Hydrogen Atom transfer (HAT) catalysis (Figure 1).

**Figure 1:**
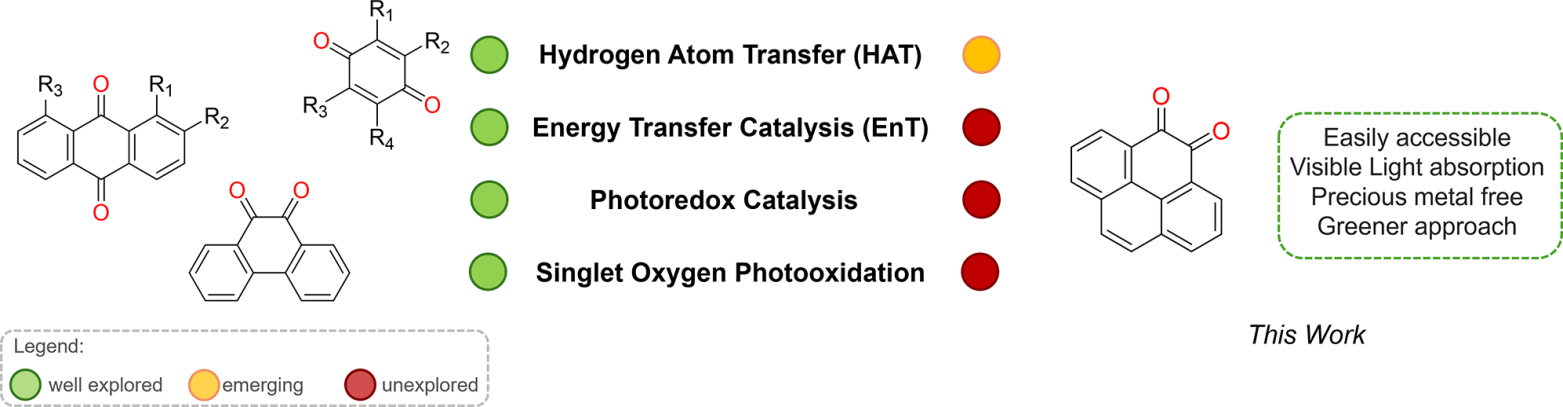
Overview of quinones as photocatalysts.

## Singlet Oxygen-Mediated Photooxidation

Singlet oxygen (^1^O_2_) is a highly reactive species photochemically produced by the combined use of a photosensitizer and ultraviolet or visible light after a singlet-to-triplet intersystem crossing. The energy of the triplet state is then transferred to the triplet ground state of molecular oxygen, generating singlet oxygen.

One of the most important examples of singlet oxygen in organic synthesis is the photooxidation of furan-based compounds.^41^ Particularly, the photooxidation of furfural combines the power of photocatalysis with biomass valorization and has been used for a variety of important products, including active pharmaceutical ingredients^42–44^ and biopolymers.^45,46^

Therefore, we began our investigation by testing PQ as a photocatalyst for the oxidation of furfural. For comparison, we evaluated other quinones previously used in singlet oxygen photosensitization: anthraquinone (AQ), alizarin (AZ), and 1,8-dihydroxyanthraquinone (1,8-DHAQ). AQ was chosen for its structural similarity to PQ, while alizarin and 1,8-DHAQ were selected due to their comparable visible-light absorption profiles (see Figure S5).

Reactions were carried out in MeOH using a 3D-printed reactor^47^ equipped with 440 nm Kessil Lamps. The reaction was monitored by Gas Chromatography with a flame ionization detector (GC-FID), and the results are shown in Figure 2. The reaction reaches full conversion after approximately 2 h of irradiation with PQ and around 3 h with AQ, showing that although AQ can be used to generate singlet oxygen with blue LEDs, its low visible absorption hinders catalyst efficiency. For Alizarin and 1,8-DHAQ, conversion of furfural was also observed; however, instead of selectively forming the desired product **1b**, a mixture of products was obtained (see GC-FID data in Figures S1–S4).

**Figure 2:**
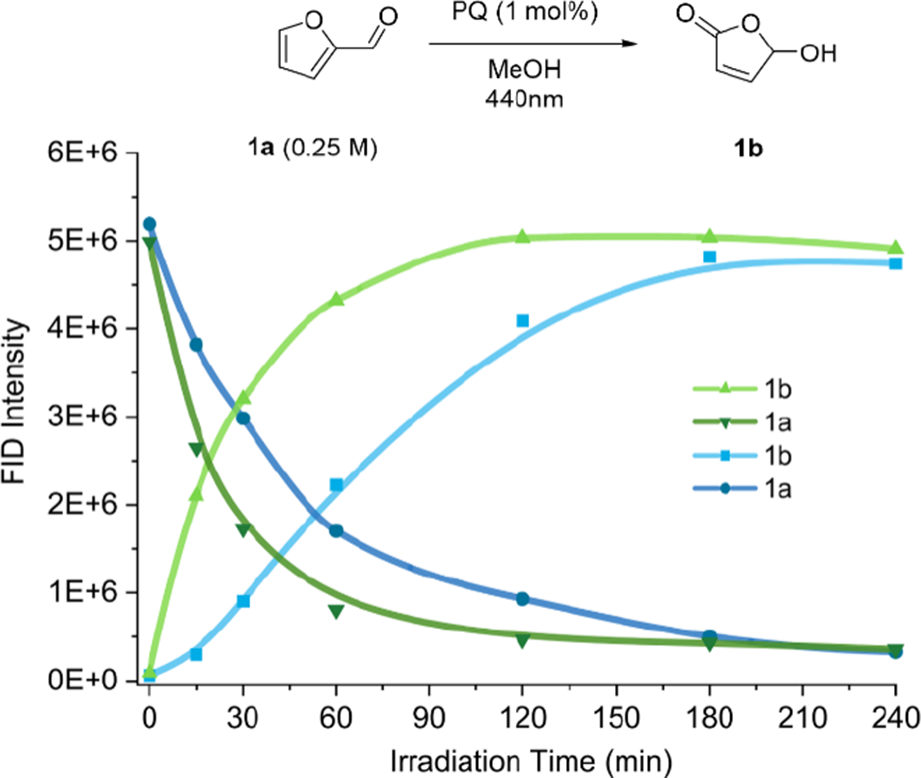
Reaction kinetics monitored by GC-FID showing the conversion of **1a** into **1b** over time using PQ (green) and AQ (blue).

We explored a set of different conditions for this reaction, and the results are shown in Table 1. When white light was used instead of blue LEDs, the yield dropped to 61% after 4 h (Table 1, entry 5). A similar decrease was observed when air was used instead of oxygen (entry 6). Negligible conversion occurred in the absence of either light or photocatalyst, confirming the photomediated nature of the oxidation.

**Table 1:** Optimization of Photooxidation of Furfural (**1a**) Using Pyrene-4,5-dione (PQ) under Visible Light^a^

entry	alteration	yield (%)^b^
1	none	93
2	AQ	77 (91^c^)
3	1,8-DHAQ	<5
4	Alizarin	<5
5^c^	white instead of 440 nm	61
6	air instead of O_2_	32
7	no light	--
8	no catalyst	--

a Conditions: Furfural (0.25 M); Photocatalyst (1 mol%); 4 mL MeOH; 440 nm Kessil Lamps; 2 h; room temperature.

b NMR yields.

c 4 h of irradiation instead of 2 h.

Photooxygenation reactions with singlet oxygen are versatile, selective, and atom-economical transformations. To verify if PQ could be an efficient catalyst for the oxidation of different substrates, a series of reactions was conducted (Figure 3).

**Figure 3:**
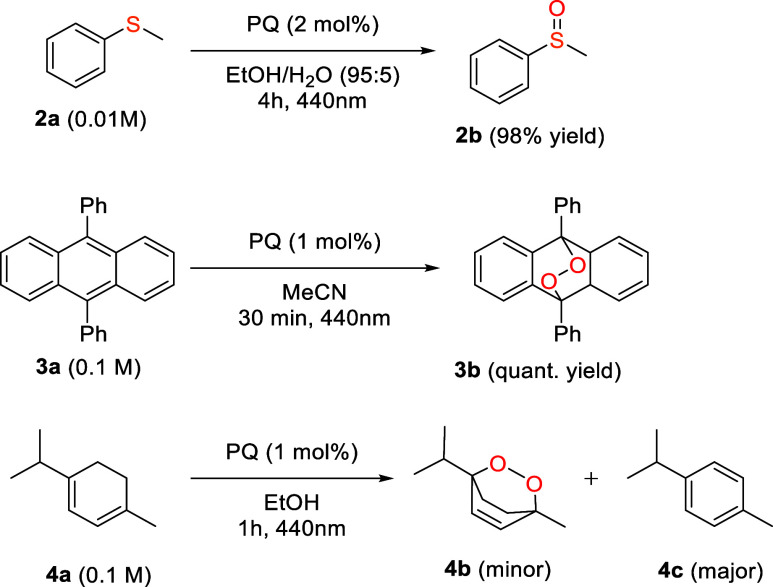
Singlet oxygen-mediated photooxidation reactions using PQ as a photocatalyst.

We first tested the oxidation of thioanisole to the corresponding sulfoxide, a class of compounds widely used as intermediates in pharmaceuticals, agrochemicals, and asymmetric synthesis.^48–52^ Irradiation in the presence of PQ (2 mol%) yielded methyl phenyl sulfoxide (**2b**) in 98% yield after 4 h, with no detectable side products (Figure 3).

9,10-Diphenylanthracene (DPA) is known to form endoperoxides upon reaction with singlet oxygen.^53^ Due to the thermal reversibility of this transformation, DPA endoperoxides have recently attracted interest as reversible photochromic and energy storage materials.^54–56^ Therefore, we verified whether endoperoxide **3b** could be formed by irradiating DPA with PQ as a photosensitizer. The photooxidation was monitored every 10 min by UV–vis spectroscopy (Figure S6), and full conversion was observed after only 30 min. Remarkably, the desired endoperoxide **3b** was obtained as the main product in quantitative yields.

Finally, we attempted the catalyzed photooxidation of α-terpinene (**4a**) to anthelmintic drug ascaridole (**4b**). To our surprise, after 1 h, full conversion was observed; however, ascaridole was only observed as a minor product. The main product observed was *p*-cymene (**4c**), with a concentration as high as 25 times that of ascaridole (Figure ESI for crude NMRs). We believe that this is due to an initial electron transfer followed by a proton transfer between PQ and **4a**, similar to what was previously reported for anthraquinone-2-carboxylic acid.^57^ Alternatively, the quinone radical anion formed by the initial electron transfer could undergo back electron transfer with oxygen, generating the superoxide radical anion (O_2_^·–^), which has been reported to be involved in the *p-*cymene formation from α-terpinene.^58^

We also attempted to obtain the Achmatowicz pyranone product from furfuryl alcohol using sodium persulfate as the oxidant instead of air. However, no product was detected and the furfuryl alcohol was recovered, indicating that although PQ is an effective singlet oxygen photosensitizer, it may not be able to photosensitize the formation of the sulfate radical anion.

Our investigation demonstrates that pyrene-4,5-dione (PQ) is an efficient singlet oxygen photosensitizer for visible-light-driven photooxidation reactions. These results highlight PQ’s potential as a sustainable and efficient photocatalyst for diverse singlet oxygen-mediated transformations, enabling applications in biomass valorisation, pharmaceutical synthesis, and the development of functional materials.

## Energy Transfer Catalysis (EnT)

Triplet–triplet energy transfer catalysis (EnT) has been attracting interest as a strategy for synthesizing organic motifs in a mild, selective, and sustainable manner. Since singlet oxygen is formed through a triplet energy transfer mechanism, we decided to investigate whether PQ could also engage in energy transfer with organic substrates. One of the most important examples of energy transfer in organic synthesis is the [2 + 2] photochemical cycloaddition. This reaction class is particularly attractive for its rapid assembly of complex molecular structures with high regio- and stereoselectivity.^59^ Therefore, we investigated the intermolecular [2 + 2] cycloaddition reactions of 3-ylideneoxindoles (**5a**),^60^ to verify whether PQ could act as an organic alternative to the precious metal-based Ru­(bpy)_3_Cl_2_ catalyst.

The irradiation of **5a** in the presence of PQ (2 mol%) led to the formation of the desired [2 + 2] cycloaddition product in excellent yield after 18 h of irradiation. The reaction time could be decreased by increasing the catalyst loading (Table 2, entry 2). Slightly lower yields were observed when DMF or DMSO were used as a solvent (Table 2, entries 3–4). Notably, the reaction gave a yield comparable to that observed when Ru­(bpy)_3_Cl_2_ was used as the photocatalyst (Table 2, entry 5). We found that the reaction is sensitive to oxygen; in the presence of air, no product was formed (Table 2, entry 6). No product was observed in the absence of either light or photocatalyst (Table 2, entries 7–8).

**Table 2:**
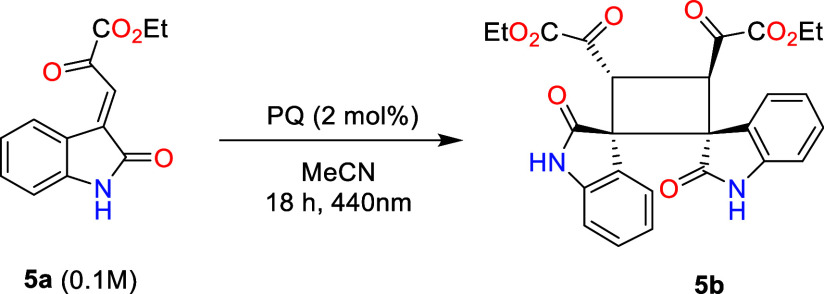
Optimization of the [2 + 2] Cycloaddition of **5a** Using PQ as Photocatalyst under Visible Light Irradiation

entry	alteration	yield (%)^a^
1	none	78
2	10 mol%, 6 h	60
3	DMF	76
4	DMSO	69
5	Ru(bpy)_3_Cl_2_ instead of PQ	74
6	air instead of argon	--
7	no light	--
8	no photocatalyst	--

a Isolated yield.

Tan and co-workers^61^ reported that anthraquinone (AQ) can engage with Selectfluor via triplet–triplet energy transfer to selectively fluorinate the most distal secondary C–H bond relative to an electron-withdrawing group within an alkyl chain. Although the reaction was performed under white light, it required reaction times longer than 24 h. Given the importance of fluorinated compounds in medicinal chemistry, we investigated whether PQ could act as a catalyst for this fluorination reaction, and whether its better absorption in the visible region could accelerate the process.

Therefore, we irradiated benzyl pentanoate (**6a**) in the presence of PQ (5 mol%) using Selectfluor as the fluorine source. Within 6 h of irradiation, we observed >80% conversion of **6a** and formation of fluorinated compounds at positions 4 (**6b**, 52%), 3 (11%), and 1 (8%), showing a clear preference for fluorination at position 4 (Table 3, entry 1). No substitution was observed at the position 2 or at terminal methyl group (position 5). Similar to the [2 + 2] cycloaddition, the reaction was sensitive to oxygen: when performed under air, only trace conversion was detected (Table 3, entry 3). Use of AQ as a photocatalyst gave a lower yield, likely due to its poor absorption at 440 nm (Table 3, entry 4), highlighting the advantage of PQ in harvesting visible light for photochemical transformations. In addition, the absence of light, the photocatalyst, or Selectfluor resulted in no significant conversion (Table 3, entries 5–7).

**Table 3:**
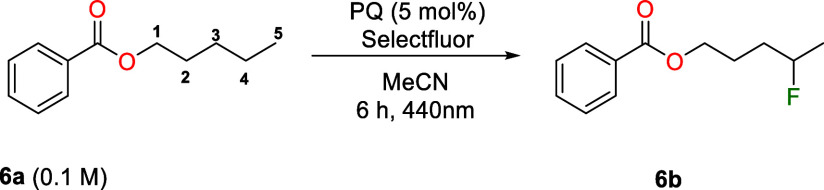
PQ-Catalyzed C–H Fluorination of Benzyl Pentanoate via Energy Transfer with Selectfluor

entry	alteration	yield (%)^a^
1	none	52
2	18 h	58
3	air instead of argon	--
4	AQ instead of PQ	19^b^
5	no light	--
6	no photocatalyst	--
7	no Selectfluor	--

a Isolated yield.

b GC yield.

These results demonstrate that PQ is an effective triplet energy transfer catalyst under visible light, enabling key transformations such as [2 + 2] cycloadditions and C–H fluorination. This highlights its potential as a sustainable alternative to metal-based EnT photocatalysts.

## Electron Transfer Reactions

In addition to energy transfer, excited states are also known to engage in electron transfer, leading to the so-called photoredox catalysis. Therefore, we investigated the ability of PQ to participate in electron transfer and function as a photoredox catalyst.

Carbon–carbon bond-forming reactions via photoredox catalysis have become a cornerstone of modern organic synthesis due to their operational simplicity and ability to generate complex structures under mild, sustainable conditions.^62^ In particular, reductive coupling reactions, such as the cross-coupling of aryl halides with π-systems, have emerged as powerful strategies for C–C bond formation without the need for preactivated organometallic reagents.^63^ The generation of aryl radicals from aryl halides enables subsequent coupling with various partners, including alkenes and electron-rich arenes, providing access to valuable structural motifs.

It has been previously reported that 1,8-dihydroxyanthraquinone undergoes electron transfer with triethylamine (TEA), forming semiquinone radical anion, and that this species can then activate carbon–halogen bonds of (hetero)­aryl halides.^24^ Related studies have shown that KOtBu can photoreduce PQ to generate a persistent semiquinonate radical.^37^ As PQ is also known to form the semiquinone radical anion when irradiated with TEA,^34^ without the need for a moisture sensitive strong inorganic base, we investigated whether PQ in the presence of TEA could enable C-X bond activation. We began by studying the reduction of aryl halides through the irradiation of 4-bromoacetophenone (**7a**) in the presence of PQ (Figure 4). The reaction reached 67% yield of **7b** after 12 h of irradiation. Notably, extending the irradiation time to 48 h increased the yield by a further 10%, resulting in full conversion and an 78% isolated yield.

**Figure 4:**
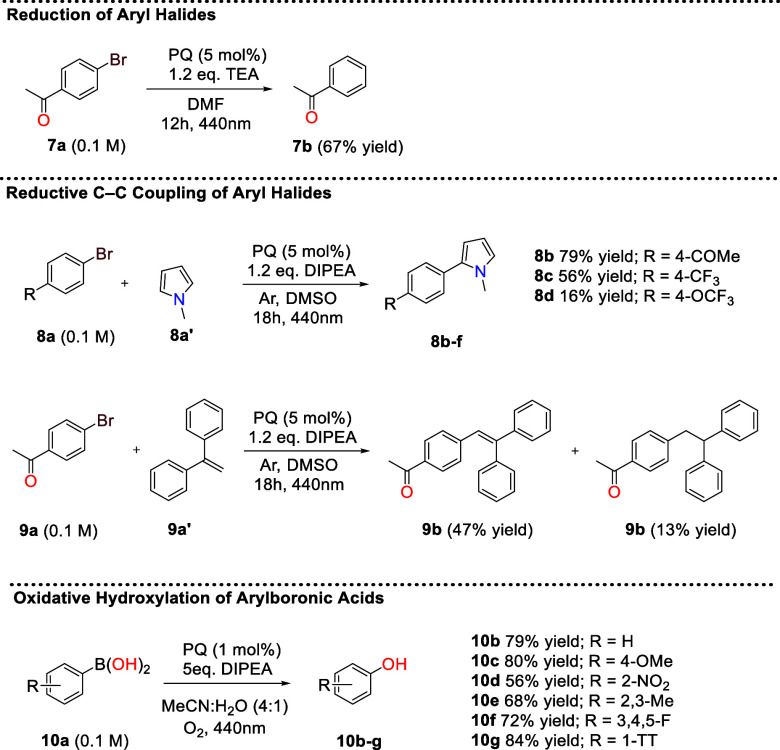
Photoredox transformations catalyzed by pyrene-4,5-dione (PQ) under visible light. Top. *NMR yields are shown for reductive reactions, and GC yields are shown for the oxidative hydroxylation reaction.

Photoredox catalytic SET reduction reactions are reported to proceed via a radical mechanism. As a result, the aryl radicals can react with arenes and unsaturated double bonds to give C–C bond-forming products through photochemical reductive C–H activation.^64^ Therefore, reaction mixtures containing aryl halides (**8a**) were irradiated in the presence of *N-*methylpyrrole (**8a′**) and 1,1-diphenylethylene (**9a′**).

The reaction between 4-bromoacetophenone and *N*-methylpyrrole in the presence of PQ led to the desired C–C coupling product **8b** in good yield (79%). Fluorinated aryl motifs are widely used in pharmaceutical design because fluorine substitution can modulate potency, metabolic stability, and physicochemical properties.^65^ Consequently, demonstrating compatibility with fluorinated aryl halides highlights the practical relevance of this coupling method for medicinal chemistry applications. Therefore, substrates bearing fluorinated substituents were evaluated, and the reaction showed moderate tolerance to the fluorinated groups tested (Figure 4, compounds **8c–8d**). When 1,1-diphenylethylene (**9a**), a known radical scavenger, was used, coupling products **9b** and **9c** were obtained in 47 and 13% isolated yields, respectively (Figure 4). 1,3,5-Trimethoxybenzene was also used as a trapping agent; however, only the reduction product was observed rather than the coupling product. It is worth noticing that activation of aryl bromides requires cleavage of an aryl C­(sp2)**−**Br bond (bond dissociation energy about 350 kJ mol^–1^).^66^ Based on prior mechanistic studies of quinone mediated reductive activation of aryl halides,^24^ the reduced quinone species formed in the presence of TEA can plausibly activate the aryl bromide either under direct irradiation or after photoexcitation of the semiquinone radical anion to access a more reducing excited state, therefore both pathways may operate in the reductive C–C coupling. Importantly, no conversion is observed in the absence of light, indicating that irradiation is required for productive bond activation. Notably, the triplet energy of PQ (∼2.0 eV, ∼190 kJ mol^–1^) is substantially lower than the aryl C­(sp2)**−**Br bond dissociation energy, suggesting that energy transfer driven bond homolysis is unlikely to be the operative pathway.

Next, we investigated the ability of PQ to perform photocatalytic oxidative coupling. Photocatalytic oxidative coupling reactions have emerged as useful methods for the construction of C–O, C–N, and C–C bonds under mild conditions, using molecular oxygen or air as green oxidants. As a model transformation, we studied the photocatalysed oxidative hydroxylation of arylboronic acids to phenols—providing straightforward access to phenolic motifs ubiquitous in natural products and pharmaceuticals. Traditional methods often require stoichiometric oxidants or transition metal catalysts. In this transformation, a superoxide radical anion is formed by the combined action of the photocatalyst and an amine^67^ which acts as the oxidizing species leading to phenol formation from arylboronic acids.^67–69^

Irradiation of phenylboronic acid (**10a**) in the presence of PQ successfully led to the formation of the desired phenol product **10b** in 79% yield. Again, the reaction demonstrated tolerance to different substrates, and good yields were observed across the series (Figure 4, compounds **10b–g**). One downside is that PQ requires a sacrificial electron donor, often an amine, because it is unable to directly reduce molecular oxygen to give the superoxide radical anion, unlike some reported hybrid materials.^58^

Overall, these results show that PQ can engage in electron transfer transformations under visible light. Under the reductive pathway, the reductive activation of 4-bromoacetophenone by PQ generates aryl radical intermediates that efficiently undergo coupling with electron-rich olefins and arenes. Under oxidative pathway, PQ promotes hydroxylation of aryl boronic acids via an oxygen-mediated pathway involving the superoxide radical anion. Together, these examples highlight PQ as a versatile metal free photocatalyst for both reductive and oxidative electron transfer reactions.

## Hydrogen Atom Transfer (HAT) Reactions

Hydrogen atom transfer (HAT) has attracted increasing attention as a complementary activation mode to energy and electron transfer, particularly for its ability to directly functionalize C–H bonds without the need for charged intermediates or activated substrates.^12^ The triplet excited state of aromatic ketones is known to undergo HAT with hydrogen donors to form a ketyl radical.^70^ Notably, ultrafast spectroscopy data have shown that PQ can undergo HAT with 2-propanol, forming a semiquinone-α-hydroxyisopropyl radical pair.^34^ The latter radical is known to react with an appropriate Michael acceptor, such as maleic acid, leading to the alkylation of electron-poor olefins.

Photocatalytic alkylation of conjugated fumaric and maleic acids with 2-propanol using benzophenone derivatives under UV irradiation has been reported to form γ-butyrolactone derivatives, versatile intermediates in the synthesis of biologically active compounds.^71,72^ However, these transformations often require high catalyst loading (0.4–3.5 equiv) and UV light (365 nm), limiting their safety, scalability and sustainability.

Therefore, we irradiated maleic acid (**11a**) with 2-propanol in the presence of PQ (5 mol%). Within 4 h of irradiation at 450 nm, full conversion of maleic acid was observed, yielding terebic acid (**11b**) in excellent yield (Figure 5). Interestingly, fumaric acid was detected at short reaction times, suggesting a photoisomerization process is operative. However, fumaric acid (**11a′**) also reacts to form the same product, thus not affecting the overall outcome of the transformation. The proposed photoredox cycle is shown in Figure 5. It has been reported that during photoinduced alcohol dehydrogenation, alcohols can form a hydrogen-bonded assembly with PQ, for which a key hydrogen-atom transfer step is rate-determining,^37^ whereas in our system the resulting α-hydroxyalkyl radical is trapped by maleic or fumaric acid, leading instead to alkylation and lactonization.

**Figure 5:**
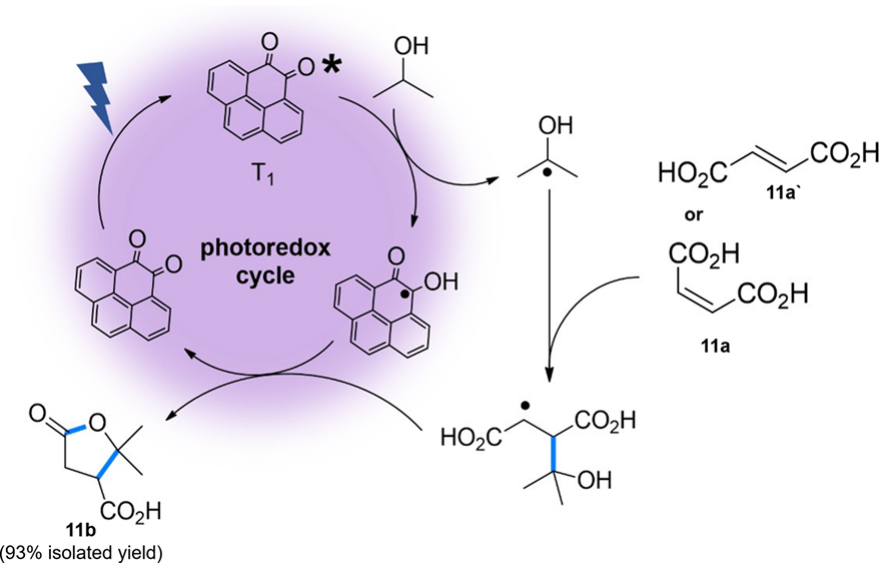
Proposed photoredox cycle for the alkylation of electron-poor olefins photocatalyzed by pyrene-4,5-dione.

We also attempted the reaction with cyclohexane, but only trace amounts of product were observed. This is likely due to the lower HAT efficiency, as 2-propanol is a significantly better hydrogen atom donor than cyclohexane.

These results demonstrate that PQ acts as an effective visible-light HAT photocatalyst, enabling the alkylation of electron-poor olefins through hydrogen abstraction under mild and metal-free conditions.

## Continuous Flow Scale-Up and Catalyst Recovery

Photochemical reactions are often challenging to scale in batch, largely because light penetration decreases as reaction volume increases and irradiation becomes nonuniform.^3^ Continuous flow provides a practical solution by using narrow reactor channels. This provides a high surface area to volume ratio, leading to more consistent photon flux, improved heat management, and more straightforward scale-up, with some examples of flow photochemistry having been demonstrated at production scale (>1 kg day^**–**1^).^73–76^ Therefore, as a proof-of-concept that PQ is a suitable organic photocatalyst under continuous flow conditions, we investigated the reductive C–C coupling between 4-bromoacetophenone and *N-*methylpyrrole in flow.

The results of the continuous flow experiments are summarized in Table 4. Varying the residence time from 10 to 120 min led to a clear increase in reaction yield, increasing from 8% at 10 min to 15% at 20 min, 45% at 60 min, and reaching 85% at 120 min (Table 4, entries 1 to 4), which is slightly higher than the yields observed in batch. Across the range of residence times, the productivity remained broadly constant, decreasing only slightly at longer residence times from about 0.19 to 0.17 mmol h^–1^. This corresponds to a space time yield (STY) of approximately 0.043 mmol h^–1^ mL^–1^ (based on the reactor volume used), giving a productivity per unit reactor volume that is comparable to related photochemical transformations reported in the literature. Additionally, numbering up two 5 mL coils at a 120 min residence time (Table 4, entry 6) maintained a similar yield (83%) while increasing the throughput to 0.41 mmol h^–1^, which gives an extrapolated productivity of 1.9 g day^–1^, while keeping a similar STY (0.041 mmol h^–1^ mL^–1^). In the numbering up experiment, an extended run was performed to collect 60 mL of reaction mixture that was then purified to give 0.94 g of the coupled product in 78% isolated yield (83% yield by GC).

**Table 4:** Yield and Productivity for PQ-Catalyzed Reductive C–C Coupling in Continuous Flow Conditions^a^

entry	res. time (min)	yield (%)^b^	produc. (mmol h^–1^)
1	10	8	0.19
2	20	15	0.18
3	60	45	0.18
4	120	85	0.17
5^c^	120	82	0.16
6^d^	120	83	0.41

a Reaction Condition: 4-bromoacetophenone (0.1M); PQ (5 mol%); *N-*methylpyrrole (10 equiv.); DMSO as solvent; 4 mL coiled Reactor; 440 nm Kessil Lamps; Room Temperature (fan cooled).

b GC yields.

c Reaction was performed with recovered catalysts.

d Reaction was performed on a 10 mL reactor by numbering up two 5 mL coils.

Organic photocatalysts present a greener approach compared to precious metal-based systems such as Ru and Ir complexes. Despite this, catalyst recovery and recycling remain major economic and environmental concerns in process chemistry.^77^ Therefore, we also explored a laboratory strategy for recovering the PQ photocatalyst.

During our purification efforts, we observed a distinct orange band at the top of the silica column used for workup. By extending the elution gradient, we recovered approximately 85–90% of the catalyst when performing the benzyl pentanoate fluorination (EnT), the C–C bond-forming reaction (photoredox), and the alkylation of conjugated fumaric acid (HAT). The recovered PQ showed no evidence of degradation or structural changes (see ESI for NMR). The recovered PQ was typically recombined with the nonused PQ. However, to assess the reusability of the recovered photocatalyst alone, the C–C bond-forming reaction between 4-bromoacetophenone and *N-*methylpyrrole (**8a**) was performed in flow using only recovered PQ (Table 4, entry 5), and the reaction gave a similar yield (82%) compared to nonused PQ (Table 4, entry 4). It is worth noticing that PQ is poorly soluble in nonpolar solvents such as hexane, which limits the use of liquid injection during flash chromatography and makes recovery impractical when liquid–liquid extraction with an apolar solvent is performed during workup. Currently, alternative recovery methods are under consideration to minimize solvent use, including heterogenisation strategies,^78,79^ and membrane separation technologies.

## Conclusions and Perspectives

Pyrene-4,5-dione (PQ) has been shown to function as a versatile, metal-free, visible-light organic photocatalyst across four distinct photochemical reaction classes: photooxidation, energy transfer, photoredox, and hydrogen atom transfer (HAT). PQ enabled a range of synthetically relevant transformations, including singlet oxygen oxidations, [2 + 2] cycloadditions, C–H fluorination, reductive C–C coupling, and HAT-driven alkylation. In each case, the reactions proceeded under metal-free conditions using commercially available blue LEDs and a 3D-printed reactor setup, leading to greener and safer outputs for photocatalysis.

As a proof-of-concept for scalability, the reductive C–C coupling was translated to continuous flow, delivering up to 85% yield at 0.17 mmol h^–1^ in a 4 mL reactor, and 83% yield at 0.41 mmol h^–1^ upon numbering-up to a 10 mL reactor. PQ could be recovered in typically 85–90% mass recovery from reaction mixtures by standard chromatographic methods, with no evidence of structural change or degradation. Recovered PQ was reused on a recycle experiment in continuous flow for the reductive C–C coupling, which delivered a comparable yield to reactions run with nonused PQ. This combination of reusability, broad applicability, and operational stability highlights PQ as a practical photocatalyst for sustainable synthetic applications.

## Experimental Methods

### General

Photochemical reaction experiments were performed in a 3D-printed reactor^47^ printed in black PETG (Ultimaker PETG Black, item code 1633) using an UltiMaker S5 3D printer. The reactor was equipped with two 440 nm Kessil Lamps (PR160L, 45 W, https://kessil.com/products/science_PR160L.php). The lamp spectrum is distributed between 410 and 470 nm and centered on 440 nm. Spectra can be found on the supplier page. The provided average intensity is 399 mW cm^–2^ (measured from 1 cm distance). Reactions were performed using the 8 mL vial holder using borosilicate glass 8 mL vials (Waters UPLC vial with cap and PTFE/Silicone septum) containing a stir bar. Cooling was provided by two Sunon axial fans (RS Components, 12 V DC, 24.9 cfm, 6.12 W, IP20, 40 × 40 × 28 mm) coupled to the 3D-printed reactor, without a heat sink. For flow experiments, the coil holder reported by Schiel and co-workers was modified from PETG to stainless steel. The stainless steel block was coiled with 1/16” PTFE tubing and connected to HPLC pumps (Teledyne M1 Class Single Piston Pump; Eccentric Drive; 0.0–40.0 mL min^–1^; 500 psi; Stainless Steel Fluid Path) using appropriate IDEX fittings for reactant and solvent delivery.

### Synthesis

#### Furfural Photooxidation

Furfural **1a** (0.25 M, 83 μL, 1 mmol), photocatalyst (PQ, AQ, Alizarin, or 1,8-DHAQ; 1 mol%), and MeOH (4 mL) were added to an 8 mL vial (Waters UPLC vial with cap and PTFE/Silicone septum) containing a stir bar. The vial was sealed, and the reaction mixture was bubbled with O_2_ using a balloon throughout the reaction time. The vial was placed in a 3D-printed parallel reactor and stirred under blue LED irradiation (2 × Kessil PR160L, 440 nm) for 4 h. Reaction progress was monitored by GC-FID until complete consumption of furfural. The solvent was evaporated, and the crude product was purified by flash chromatography (Teledyne CombiFlash; hexane:EtOAc, gradient 95:5 to 30:70) to give a white solid. **1b**: ^1^H NMR (400 MHz, Chloroform-d) δ 7.23 (dd, *J* = 5.7, 1.2 Hz, 1H), 6.26 (dd, *J* = 5.7, 1.2 Hz, 1H), 5.88 (t, *J* = 1.2 Hz, 1H), 3.60 (s, 3H). Characterization data in agreement with the literature data.^45^

#### Sulfide Photooxidation

In a glass vial containing thioanisole **2a** (0.1 M, 47 μL, 0.4 mmol) and PQ (0.0027 g, 0.01 mmol, 2 mol%), 4 mL of EtOH/H_2_O (95:5) was added. The reaction was carried out in a 3D-printed parallel reactor under blue LED irradiation (Kessil PR160L, 440 nm) for 4 h, with oxygen bubbled continuously via balloon. The reaction was monitored by GC-MS. The solvent was evaporated, and the crude product was purified by flash chromatography (Teledyne CombiFlash; hexane:EtOAc, gradient 100:0 to 50:50) to give a low-melting-point white solid (55 mg, 0.39 mmol, 98% yield).

**2b**: ^1^H NMR (400 MHz, Chloroform-d) δ 7.66–7.60 (m, 2H), 7.55–7.45 (m, 3H), 2.70 (s, 3H). ^13^C­{^1^H} NMR (101 MHz, Chloroform-d) δ 144.6, 129.9, 128.2, 122.4, 42.9. GC-MS: rt: 8.35 min; mz: 109, 125, 140.0 [M]. Characterization data in agreement with the literature data.^52^

#### DPA Photooxidation

In a glass vial containing 9,10-diphenylanthracene **3a** (DPA, 0.1 M, 0.132 g, 0.4 mmol) and PQ (1 mol%, 1 mg), 4 mL of MeCN was added. The reaction mixture was transferred to a 3D-printed parallel reactor and stirred under blue LED irradiation (Kessil PR160L, 440 nm) for 2 h. During the irradiation, a flow of oxygen was bubbled through the solution using a balloon to ensure sufficient oxygen availability. The DPA concentration was measured using UV–vis spectroscopy, and the consumption of DPA was calculated by monitoring the decrease in absorption in the 380–450 nm region, and the yield was confirmed by NMR.

**3b**: ^1^H NMR (500 MHz, Chloroform-*d*) δ 7.73–7.68 (m, 4H), 7.66–7.60 (m, 4H), 7.57–7.53 (m, 2H), 7.24–7.14 (m, 8H). ^13^C­{^1^H} NMR (126 MHz, Chloroform-*d*) δ 140.3 133.1, 128.4, 128.4, 127.7, 127.6, 123.6, 84.2. Characterization data in agreement with the literature data.^56^

#### α-Terpinene Photooxidation

In a glass vial containing α-terpinene **4a** (0.1 M, 65 μL, 0.4 mmol) and PQ (1 mol%, 1 mg), 4 mL of EtOH was added. The reaction mixture was transferred to a 3D-printed parallel reactor and stirred under blue LED irradiation (Kessil PR160L, 440 nm) for 2 h. During the irradiation, a flow of oxygen was bubbled through the solution using a balloon to ensure sufficient oxygen availability. The α-terpinene concentration was measured using NMR spectroscopy.

#### [2 + 2] Photochemical Cycloaddition

Reaction was adapted from Zuo et al.^60^ To a mixture of (*E*)-ethyl 2-(1,5-dimethyl-2-oxoindolin-3-ylidene)­acetate **5a** (0.1 M, 98 mg, 0.4 mmol) and PQ (2 mol%, 1.8 mg) in an 8 mL vial, MeCN (4.0 mL) was added. The vial was sealed, and air was replaced by bubbling the reaction mixture with purified argon for 30 min. The mixture was transferred to a 3D-printed parallel reactor and stirred under blue LED irradiation (Kessil PR160L, 440 nm) for 18 h. After complete consumption of **5a** (monitored by TLC analysis), H_2_O (10 mL) was added to the reaction mixture. The resulting mixture was extracted with Et_2_O (3 × 10 mL), and the combined organic layers were dried over Na_2_SO_4_. The solvent was removed under vacuum, and the residue was purified by flash chromatography (CombiFlash+, SilicaGold Column; hexane:EtOAc 100:0 to 40:60) to give an off-white solid (77 mg, 0.16 mmol, 78% yield).

**5b**: ^1^H NMR (500 MHz, Chloroform-*d*) δ 8.10 (s, 2H), 7.67 (d, *J* = 7.7 Hz, 2H), 7.18–7.11 (m, 2H), 6.98 (t, *J* = 7.6 Hz, 2H), 6.67 (d, *J* = 7.7 Hz, 2H), 4.51 (s, 2H), 3.88 (ddd, *J* = 60.0, 10.7, 7.0 Hz, 4H), 0.76 (t, *J* = 7.0 Hz, 6H). ^13^C­{^1^H} NMR (126 MHz, Chloroform-*d*) δ 174.8, 169.0, 140.8, 129.3, 128.5, 123.6, 121.2, 109.6, 61.0, 54.9, 43.0, 13.8. Characterization data in agreement with the literature data.^60^

#### Photochemical C–H Fluorination

Reaction was adapted from Kee et al.^61^ In a glass vial containing Selectfluor (0.067 M, 96 mg, 0.27 mmol), pentyl benzoate **6a** (0.1 M, 78 μL, 0.4 mmol, 1.5 equiv), and PQ (5 mol%, 4.7 mg, 0.02 mmol), 4 mL of MeCN was added. The vial was sealed, and air was replaced by bubbling the reaction mixture with purified argon for 30 min. The reaction mixture was transferred to a 3D-printed parallel reactor and stirred under blue LED irradiation (Kessil PR160L, 440 nm) for 6 h. After irradiation, 30 mL of diethyl ether (inhibitor-free) was added, causing immediate precipitation of unreacted Selectfluor and its byproduct salts. The mixture was filtered, and the residue was rinsed with 3 × 10 mL of diethyl ether (inhibitor-free). The solvent was evaporated, and the residue was purified by flash chromatography (CombiFlash+, SilicaGold Column; hexane:Et_2_O 100:0 to 90:10, then up to 40:60 for catalyst recovery) to give a white solid (29 mg, 0.14 mmol, 52% yield).

**6b**: ^1^H NMR (400 MHz, Chloroform-*d*) δ 8.14–7.94 (m, 2H), 7.62–7.51 (m, 1H), 7.51–7.38 (m, 2H), 4.93–4.57 (m, 1H), 4.46–4.26 (m, 2H), 2.04–1.62 (m, 4H), 1.36 (dd, *J* = 23.8, 6.2 Hz, 3H). ^19^F NMR (376 MHz, Chloroform-*d*) δ −173.29. ^13^C­{^1^H} NMR (101 MHz, Chloroform-*d*) δ 166.7, 133.1, 130.5, 129.7, 128.5, 91.4, 89.7, 64.7, 33.7, 33.5, 24.7, 24.7, 21.3, 21.0. GC-MS: rt: 8.81 min; mz: 105.0, 122.0, 135.0_(w)_, 181.0_(w)_, 210.1_(w)_ [M]. Characterization data in agreement with the literature.^61^

#### Photoredox Reductive Reactions

Reactions were adapted from Bardagi et al.^24^

##### General Procedure for the Photoreduction of Aryl Halides

In an 8 mL glass vial containing 4-bromoacetophenone **7a** (0.1 M, 80 mg, 0.4 mmol, 1 equiv) and PQ (5 mol%, 4.9 mg), 4 mL of DMF (Acroseal, dry) was added. The vial was sealed, and air was replaced by bubbling the reaction mixture with purified argon for 15 min. Triethylamine (70 μL, 0.5 mmol 1.2 equiv) was added under argon, and the mixture was bubbled for another 15 min. The reaction mixture was transferred to a 3D-printed parallel reactor and stirred under blue LED irradiation (Kessil PR160L, 440 nm) for 12 h. Reaction progress was monitored by GC-MS. Yields of the reduction products were determined by GC with appropriate internal standards. The mixture was transferred into a separating funnel, and water (30 mL) and brine (10 mL) were added. The resulting mixture was extracted with EtOAc (3 × 25 mL), dried over MgSO_4_, filtered, and concentrated under vacuum. The residue was purified by flash chromatography (CombiFlash+, SilicaGold Column; hexane:EtOAc 100:0 to 60:40, then up to 40:60 for catalyst recovery).

Acetophenone (**7b**): clear oil (67% yield, 32 mg, 0.27 mmol). ^1^H NMR (400 MHz, Chloroform-*d*) δ 7.99–7.93 (m, 2H), 7.59–7.53 (m, 1H), 7.49–7.43 (m, 2H), 2.60 (s, 3H). ^13^C­{^1^H} NMR (101 MHz, Chloroform-*d*) δ 198.1, 137.1, 133.1, 128.5, 128.3, 26.6. GC-MS: rt: 5.73 min; mz: 77.0, 105.0 120.0 [M]. Characterization data in agreement with the literature data.^28^

##### General Procedure for Reductive C–C Coupling

In an 8 mL glass vial containing aryl halide **8a** (0.1 M, 0.4 mmol, 1 equiv) and PQ (5 mol%, typically ∼5 mg), 4 mL of DMSO was added. The vial was sealed, and air was replaced by bubbling the reaction mixture with purified argon for 15 min. Then DIPEA (85 μL, 0.49 mmol, 1.2 equiv) and the trapping reagent (10 equiv., 4 mmol; 360 μL for *N-*methylpyrrole **8a’** or 710 μL for 1,1-diphenylethene **9a’**) were added under argon, and the mixture was bubbled for another 15 min. The reaction mixture was transferred to a 3D-printed parallel reactor and stirred under blue LED irradiation (Kessil PR160L, 440 nm) for 18 h. Reaction progress was monitored by GC-MS. Yields of the products were determined by NMR using internal standards. The reaction mixture was transferred to a separating funnel, and water (30 mL) and brine (10 mL) were added. The resulting mixture was extracted with EtOAc (3 × 25 mL), dried over MgSO_4_, filtered, and concentrated in vacuo. The crude product was purified by flash chromatography (CombiFlash+, SilicaGold Column; hexane:EtOAc 100:0 to 60:40, then up to 40:60 for catalyst recovery).

**8b** (4-COMe): light yellow oil (79% yield, 62 mg, 0.31 mmol). ^1^H NMR (400 MHz, Chloroform-*d*) δ 8.12–7.90 (m, 2H), 7.61–7.36 (m, 2H), 6.91–6.67 (m, 1H), 6.34 (dd, *J* = 3.7, 1.8 Hz, 1H), 6.22 (dd, *J* = 3.6, 2.7 Hz, 1H), 3.71 (s, 3H), 2.61 (s, 3H). ^13^C­{^1^H} NMR (101 MHz, Chloroform-*D*) δ 197.7, 138.0, 135.0, 133.5, 128.7, 128.1, 125.4, 110.3, 108.5, 35.6, 26.7. GC-MS: rt: 11.60 min mz: 199.1 [M]; 184.1, 156.1. Characterization data in agreement with the literature data.^80^

**8c** (4-CF_3_): yellowish oil (55% yield, 50 mg, 0.22 mmol). ^1^H NMR (400 MHz, Acetonitrile-*d*_3_) δ 7.66 (d, *J* = 7.8 Hz, 2H), 7.55 (d, *J* = 8.0 Hz, 2H), 6.76 (d, *J* = 1.6 Hz, 1H), 6.26 (dd, *J* = 3.6, 1.7 Hz, 1H), 6.15–6.04 (m, 1H), 3.63 (s, 3H).^19^F NMR (376 MHz, Acetonitrile-*d*_3_) δ −62.71. ^13^C­{^1^H} NMR (101 MHz, Acetonitrile-*d*_3_) δ 137.3, 132.5, 128.2, 125.6, 125.5, 125.4, 125.4, 125.3, 110.0, 108.0, 35.4. GC-MS: rt: 7.89 min mz: 225.1 [M]. Characterization data in agreement with the literature data.^81^

**8d** (4-OCF_4_): clear oil (16% yield, 16 mg, 0.06 mmol). ^1^H NMR (400 MHz, Acetonitrile-*d*_3_) δ 7.47 (dd, *J* = 8.8, 3.0 Hz, 2H), 7.38–7.26 (m, 2H), 6.73 (d, *J* = 2.3 Hz, 1H), 6.23–6.13 (m, 1H), 6.14–6.02 (m, 1H), 3.62 (s, 3H). ^19^F NMR (376 MHz, Acetonitrile-*d*_3_) δ −58.55. ^13^C­{^1^H} NMR (101 MHz, Acetonitrile-*d*_3_) δ 147.7, 132.8, 132.6, 130.1, 124.7, 121.9, 121.2, 119.4, 109.2, 107.7, 34.6. CG-MS: rt: 7.70 min mz 241.0 [M]. Characterization data in agreement with the literature data.^80^

**9b**: White solid (47% yield, 56 mg, 0.19 mmol). ^1^H NMR (500 MHz, Chloroform-*d*) δ 7.72 (d, *J* = 8.5 Hz, 2H), 7.37–7.31 (m, 8H), 7.21–7.17 (m, 2H), 7.09 (d, *J* = 8.4 Hz, 2H), 6.99 (s, 1H), 2.53 (s, 3H). ^13^C­{^1^H} NMR (126 MHz, Chloroform-*d*) δ 197.7, 145.3, 143.0, 142.4, 139.9, 135.1, 130.4, 129.7, 128.9, 128.4, 128.2, 128.1, 127.9, 127.9, 127.1, 26.6. Characterization data in agreement with the literature data.^24^

**9c**: White solid (13% yield, 16 mg, 0.05 mmol). ^1^H NMR (500 MHz, Chloroform-*d*) δ 7.76 (d, *J* = 8.3 Hz, 2H), 7.27–7.22 (m, 4H), 7.21–7.14 (m, 6H), 7.08 (d, *J* = 8.3 Hz, 2H), 4.23 (t, *J* = 7.9 Hz, 1H), 3.41 (d, *J* = 7.9 Hz, 2H), 2.53 (s, 3H).^13^C­{^1^H} NMR (126 MHz, Chloroform-*D*) δ 198.1, 146.2, 144.0, 135.2, 129.4, 128.6, 128.4, 128.0, 126.5, 52.7, 42.2, 26.7. Characterization data in agreement with the literature data.^82^

1,3,5-Trimethoxybenzene as trapping agent: reduction observed, no coupling product detected.

#### Oxidative Hydroxylation of Arylboronic Acids

Reaction was adapted from Pitre et al.^67^ arylboronic acid **10a** (0.1 M, 0.4 mmol, 1 equiv), PQ (1 mol%, typically ∼1 mg), DIPEA (350 μL, 2 mmol, 5 equiv), and MeCN:H_2_O (4:1, 4 mL) were added to an 8 mL vial with a stir bar. The vial was sealed with a balloon filled with O_2_. The reaction mixture was transferred to a 3D-printed parallel reactor and stirred under blue LED irradiation (Kessil PR160L, 440 nm) for 6 h. The reaction was quenched with 5 mL of 10% HCl, extracted with Et_2_O (×3), washed with brine (×3), dried over MgSO_4_, and filtered under vacuum. The crude product was quantified by GC-FID using the respective phenol for calibration. Yields were confirmed by ^1^H NMR using 1,3,5-trimethoxybenzene as an internal standard.

Phenol (**10b**): white solid. ^1^H NMR (400 MHz, Chloroform-*d*) δ 7.33–7.24 (m, 2H), 7.02–6.93 (m, 1H), 6.92–6.84 (m, 2H), 4.39 (s, 1H).^13^C­{^1^H} NMR (101 MHz, Chloroform-*d*) δ 155.4, 129.7, 120.8, 115.3. GC-MS: rt: 4.77 min; mz: 94.1 [M], 66.1.

4-methoxyphenol (**10c**): ^1^H NMR (400 MHz, Acetonitrile-*d*_3_) δ 6.86–6.60 (m, 4H), 6.49 (s, 1H), 3.67 (s, 3H). ^13^C­{^1^H} NMR (101 MHz, Acetonitrile-*d*_3_) δ 153.3, 150.7, 115.8, 114.7.

2-nitrophenol (**10d**): yellow solid. ^1^H NMR (400 MHz, Acetonitrile-*d*_3_) δ 10.27 (s, 1H, OH), 8.07 (dd, *J* = 8.5, 1.6 Hz, 1H), 7.62 (ddd, *J* = 8.7, 7.2, 1.7 Hz, 1H), 7.14 (dd, *J* = 8.5, 1.2 Hz, 1H), 7.01 (ddd, *J* = 8.5, 7.2, 1.3 Hz, 1H). GC-MS: rt: 6.25 min; mz: 139.0 [M], 109.0, 81.1. Characterization data in agreement with the literature data.^83^

2,3-dimethylphenol (**10e**): brownish solid ^1^H NMR (400 MHz, Acetonitrile-*d*_3_) δ 6.87 (t, *J* = 7.8 Hz, 1H), 6.73–6.54 (m, 3H), 2.19 (s, 3H), 2.06 (s, 3H). GC-MS: rt: 6.45 min; mz: 122.1 [M], 107.1. Characterization data in agreement with the literature data.^84^

3,4,5-trifluorophenol (**10f**): white solid. ^1^H NMR (400 MHz, Acetonitrile-*d*_3_) δ 7.47 (s, 1H), 6.64–6.41 (m, 2H). ^19^F NMR (376 MHz, Acetonitrile-*d*_3_) δ −136.71, −175.28. ^13^C­{^1^H} NMR (101 MHz, Acetonitrile-d_3_) δ 153.0, 152.9, 152.9, 152.7, 152.6, 152.6, 152.5, 152.4, 150.2, 150.1, 150.1, 150.0, 135.3, 135.2, 135.0, 132.9, 132.8, 132.6, 100.1, 100.1, 100.0, 99.9. GC-MS: rt: 4.96 min; mz: 148.0 [M], 120.0. Characterization data in agreement with the literature data.^85^

1-thianthrenol (**10g**): off-white/beige solid. ^1^H NMR (500 MHz, Chloroform-*d*) δ 7.52–7.46 (m, 2H), 7.30–7.23 (m, 2H), 7.11 (d, *J* = 7.9 Hz, 1H), 7.03 (dd, *J* = 7.7, 1.2 Hz, 1H), 6.83 (dd, *J* = 8.0, 1.2 Hz, 1H). ^13^C­{^1^H} NMR (101 MHz, Chloroform-*d*) δ 153.8, 137.8, 136.7, 134.2, 129.0, 128.3, 127.9, 121.3, 120.7, 114.3. Characterization data in agreement with the literature data.^86^

#### HAT Reaction

Reaction was adapted from Dondi et al.^71^ In a glass vial containing maleic (or fumaric) acid (0.1 M, 48 mg, 0.4 mmol) and PQ (5 mol%, 5 mg), 4 mL of 2-propanol was added. The vial was sealed, and air was replaced by bubbling the reaction mixture with purified argon for 30 min. The mixture was transferred to a 3D-printed parallel reactor and stirred under blue LED irradiation (Kessil PR160L, 440 nm) for 6 h. Reaction progress was monitored by GC-MS. Yields of the products were determined by GC with appropriate internal standards. Water (25 mL) was added, and the mixture was extracted with Et_2_O (3 × 25 mL). The combined organic phases were dried over MgSO_4_ and concentrated under vacuum. The residue was purified by flash chromatography (CombiFlash+, SilicaGold Column; hexane:Et_2_O, 100:0 to 60:40) to give a white solid (93% yield, 60 mg, 3.8 mmol).

Terebic acid (**11b**): ^1^H NMR (400 MHz, DMSO-*d*_6_) δ 3.23 (t, *J* = 8.5 Hz, 2H), 2.83 (dd, *J* = 17.7, 8.4 Hz, 2H), 2.71 (dd, *J* = 17.6, 8.7 Hz, 2H), 1.50 (s, 6H), 1.28 (s, 6H). ^13^C­{^1^H} NMR (101 MHz, DMSO) δ 174.4, 171.8, 83.9, 49.6, 31.7, 27.8, 23.1. Characterization data in agreement with the literature data.^71^

## Supplementary Material



## Data Availability

The data underlying this study are available in the published article and its Supporting Information.

## Notes

Experimental details, GC-FID and UV–vis data, and characterization data for all compounds (PDF)
