# Supplementary material for: Pyrene-4,5-dione as a Visible-Light Organic Photocatalyst for Photooxidation, Photoredox, Energy Transfer, and Hydrogen Atom Transfer Reactions
Source: ACS Org Inorg Au. 2026 Jan 6;6(3):303–13. doi: 10.1021/acsorginorgau.5c00111 (PMC13237607; doi:10.1021/acsorginorgau.5c00111)
Supplement: Supplementary file 1 [file gg5c00111_si_001.pdf]

Pyrene-4,5-dione as a Visible-Light Organic Photocatalyst for Photooxidation, Photoredox, Energy Transfer, and Hydrogen Atom Transfer Reactions.

Rodolfo I. Teixeira[1*], Joseph P. Anslow,[1] and Nanci C. de Lucas[2]
[1]*Department of Chemical Engineering, Loughborough University, Loughborough, LE11 3TU, UK.*
[2]*Instituto de Química, Universidade Federal do Rio de Janeiro, Cidade Universitária, RJ 21941-909, Brazil.*
*r.i.teixeira@lboro.ac.uk

Keywords: photocatalysis; pyrene-4,5-dione; energy transfer; electron transfer; HAT catalysis; photooxidation.

Contents

S1. Experimental Details 2

a) General Methods 2

S2. Additional GC data 3

S3. Additional UV and Fluorescence data 5

S4. Characterisation data for compounds 6

S1. Experimental

a) General

Reagents and solvents were purchased from Fisher and used without further purification, unless otherwise described. Furfural, thioanisole, 9,10-diphenylanthracene (DPA), *(E)*-ethyl 2-(1,5-dimethyl-2-oxoindolin-3-ylidene)acetate, Selectfluor, pentyl benzoate, 4-bromoacetophenone, triethylamine (TEA), aryl halides, *N,N*-Diisopropylethylamine (DIPEA), *N*-methylpyrrole, 1,1-diphenylethene, phenylboronic acids, maleic acid, and fumaric acid were purchased from Fisher or Merck and used without purification. Pyrene-4,5-dione (PQ) was prepared following the literature procedure.[1] All solvents used were HPLC grade or higher.

Automatic flash chromatography was performed using a Teledyne ISCO CombiFlash Rf+ system, using UV detection. The methods were developed by using the scout run for defining the gradient. Hexane and ethyl acetate (EtOAc) were used as eluents.

Proton nuclear magnetic resonance (^{1}H NMR), proton-decoupled carbon nuclear magnetic resonance (^{13}C{^{1}H} NMR) spectra and 2D NMR spectra (COSY, HSQC, and HMBC) were obtained using a JEOL ECS-400 or ECZ-500 spectrometer. The ^{1}H residual signals of the solvent were used as reference ($CDCl_3$ 7.26 ppm, DMSO-d_6 2.50 ppm), as well as the ^{13}C signals of the solvent ($CDCl_3$ 77.16 ppm, DMSO-d_6 39.52 ppm). Data are represented as follows: chemical shift (δ), integration, multiplicity (s = singlet, d = doublet, t = triplet, q = quartet, dd = doublet doublet, ddd = doublet doublet doublet, dt = doublet triplet, tdt = triplet doublet triplet, m = multiplet), coupling constants (J) is in Hertz (Hz). NMR spectra were processed with MestReNova Software.

GC-FID analyses were carried out using a Shimadzu GC-2014 system equipped with a 30 m length, 0.25 mm diameter Supelco Equity-1701 column (0.25 µm particles, fused silica with polycyanopropylphenyl/polydimethylsiloxane bonded phase – intermediate polarity. A temperature gradient from 50°C to 280°C, over 15 min, then holding at 280°C for a further 5 min. Flame ionisation detection was used, with a temperature of 300°C, a 4 µL injection volume of sample (50.0 split ratio), and He as the carrier gas (column flow rate of 1.36 mL min^{-1}).

GC-MS analyses were carried out using an Agilent GC 6890N coupled with a 5975C VL MSD with triple-axis detector GC-2014 system equipped with a 30 m length, 0.25 mm diameter Supelco SPB-50 column with a 0.25 µm film thickness of fused silica with matrix active group of poly(50% diphenyl / 50% dimethyl siloxane) phase bonded phase – capillary intermediate polarity. Sample injection was performed by a 7693A autosampler using a 1 µL injection volume of sample (100.0 split ratio, split flow: 100 mL/min) with a front inlet temperature of 280°C. The oven was set with a temperature gradient from 60°C to 280°C over 10 min and a holding temperature of 280°C for a further 5 min (15 min total). Helium was used as the carrier gas at a column flow rate of 1 mL min^{-1} (8.2 psi). Mass detection (MSD) was used, with a temperature of 250°C.

S2. GC-FID data

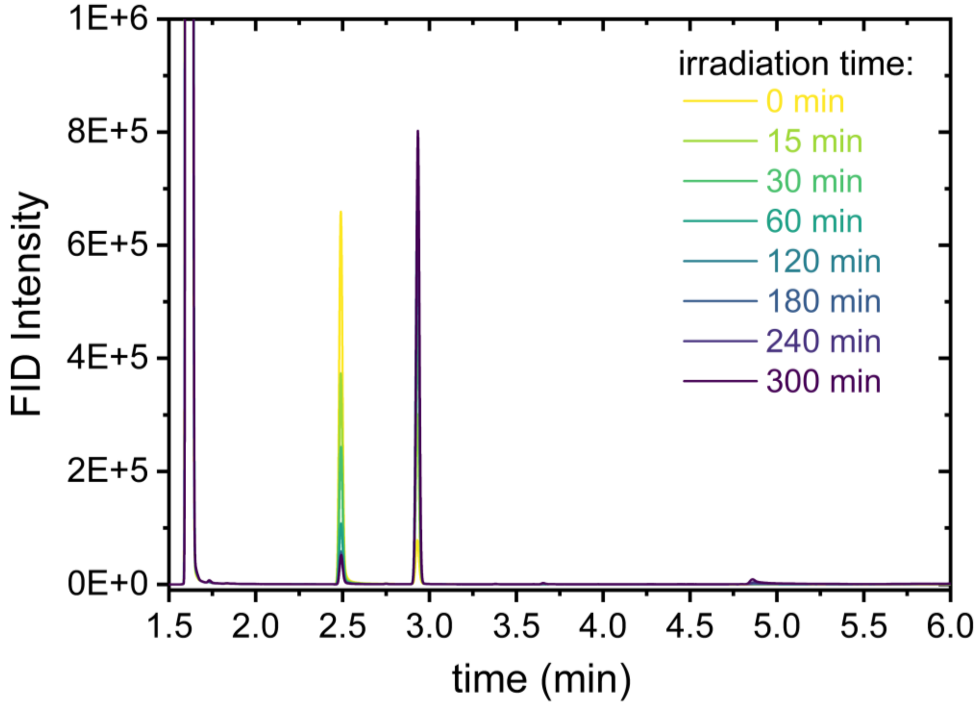

Figure S1. GC-FID monitoring of furfural oxidation using PQ as photocatalyst.

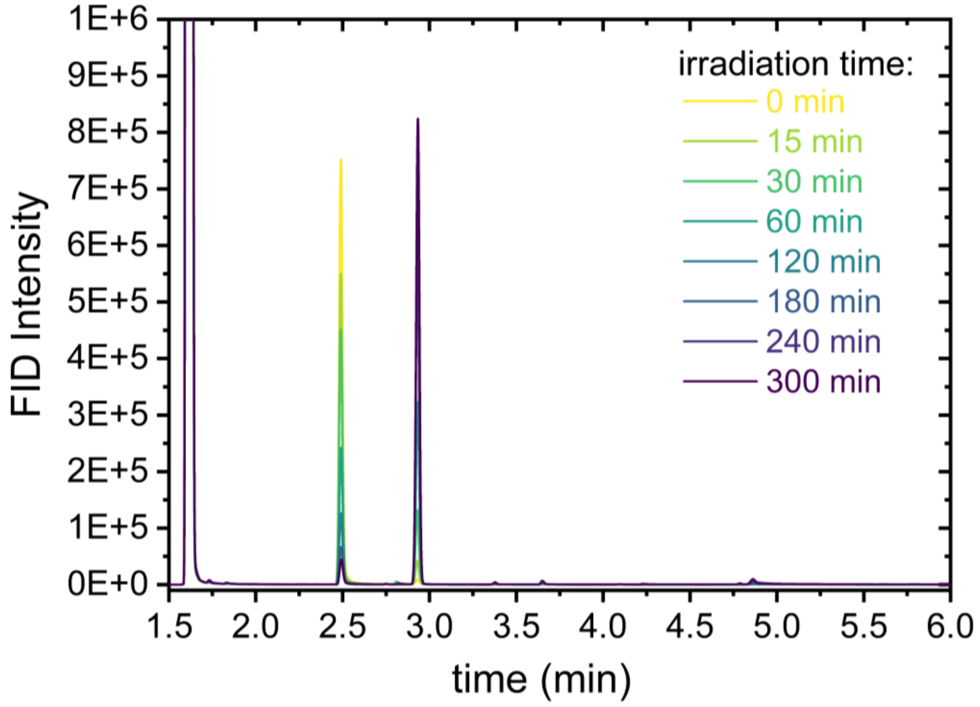

Figure S2. GC-FID monitoring of furfural oxidation using AQ as photocatalyst.

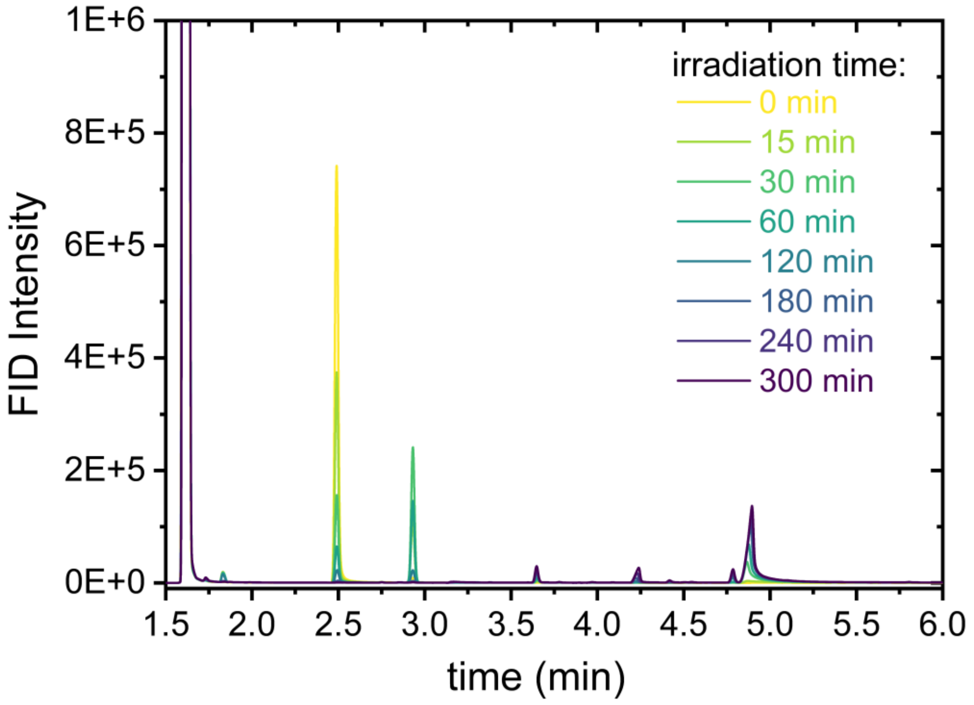

Figure S3. GC-FID monitoring of furfural oxidation using Alizarin as photocatalyst.

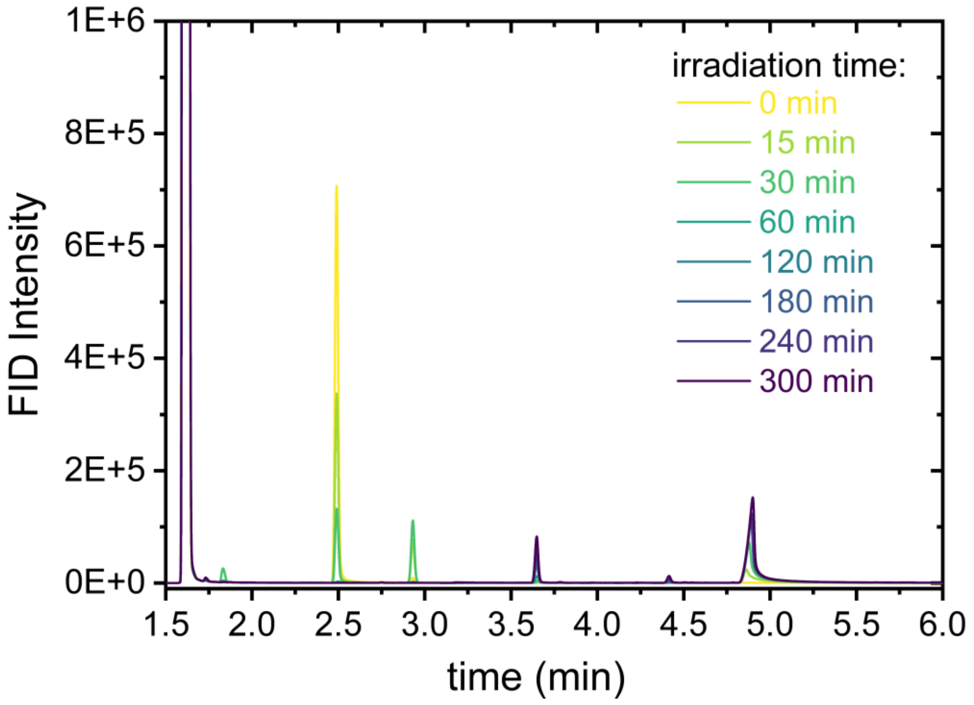

Figure S4. GC-FID monitoring of furfural oxidation using DHQ as photocatalyst.

S3. ADD UV Vis DPA

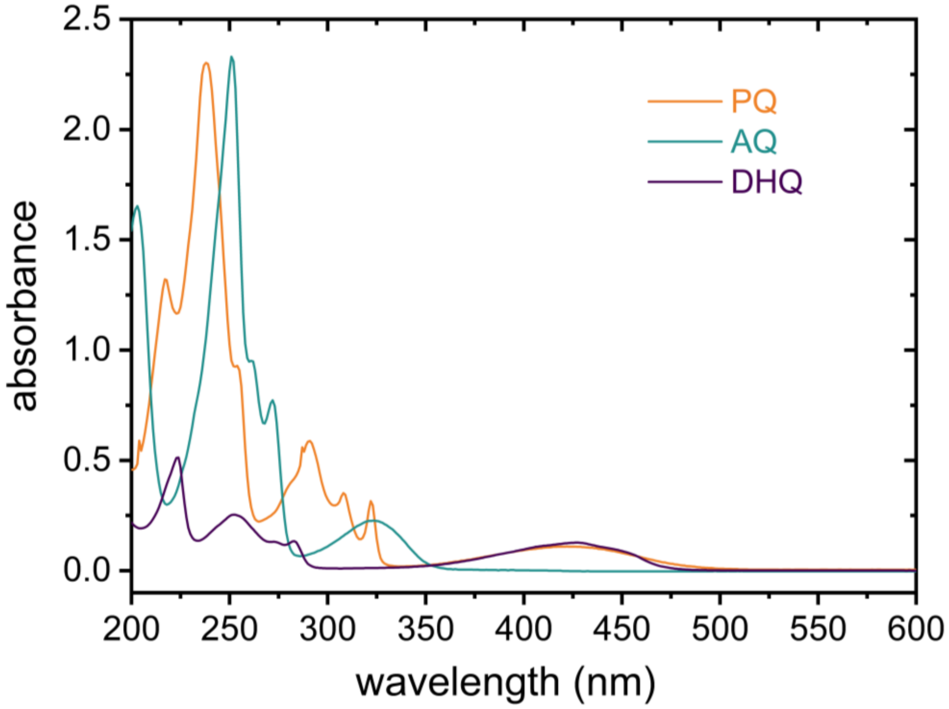

Figure S5. Absorption spectra of pyrene-4,5-diones (PQ), anthraquinone (AQ), and 1,8-dihydroxyanthraquinone (DHQ).

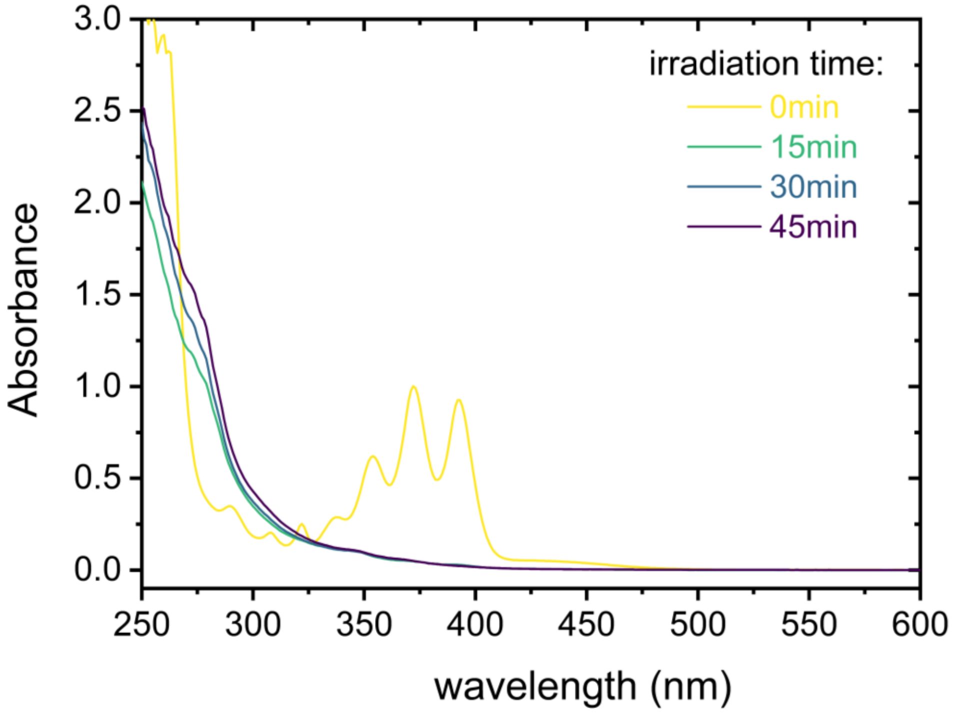

Figure S6. DPA photooxidation was monitored every 10 min using UV-Vis

S4. Characterization Data

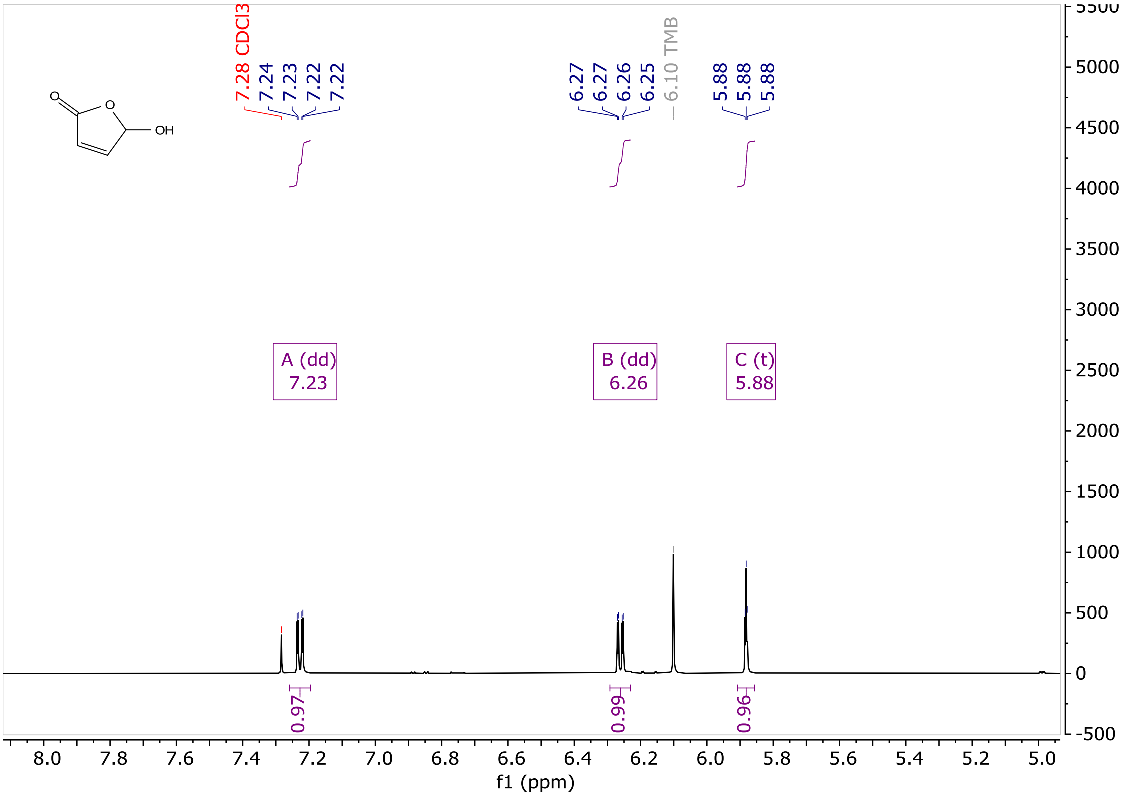

Figure S7: ^{1}H NMR ($CDCl_3$, 400MHz) spectrum of crude **1b**.

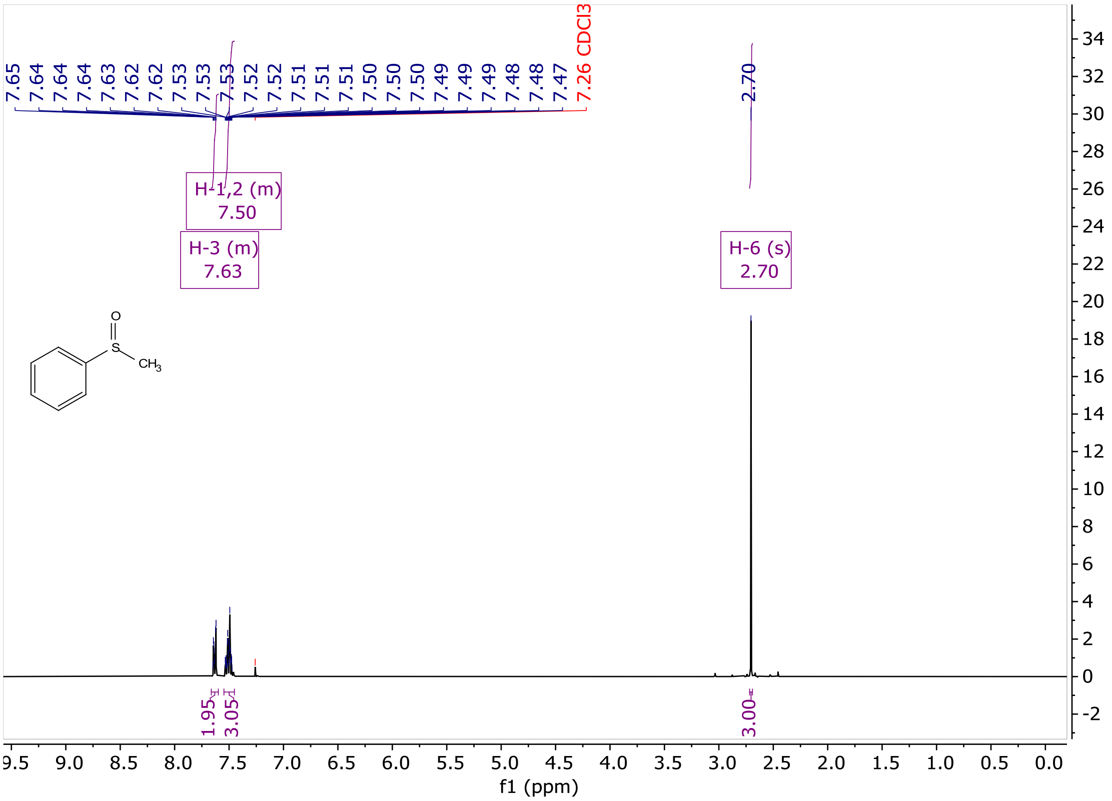

Figure S8: ^{1}H NMR ($CDCl_3$, 400MHz) spectrum of **2b**.

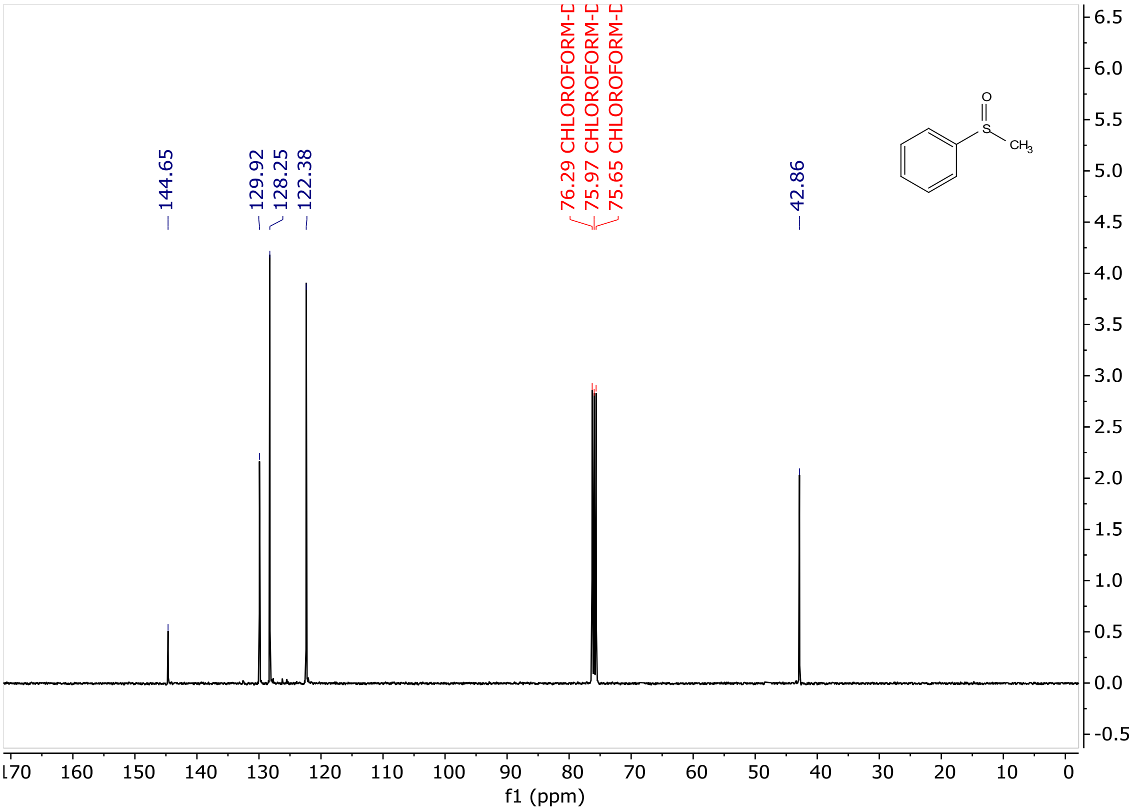

Figure S9: $^{13}C\{^1H\}$ NMR ($CDCl_3$, 101 MHz) spectrum of **2b**.

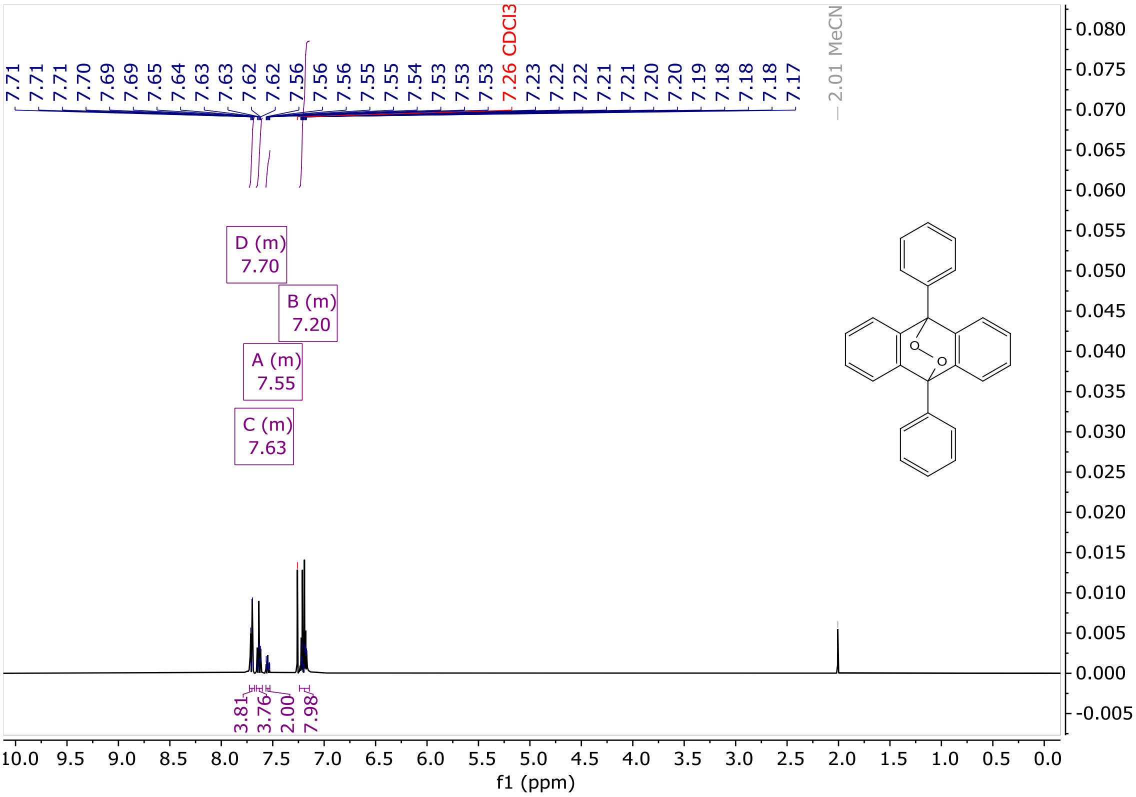

Figure S10: ^1H NMR ($CDCl_3$, 400MHz) spectrum of **3b**.

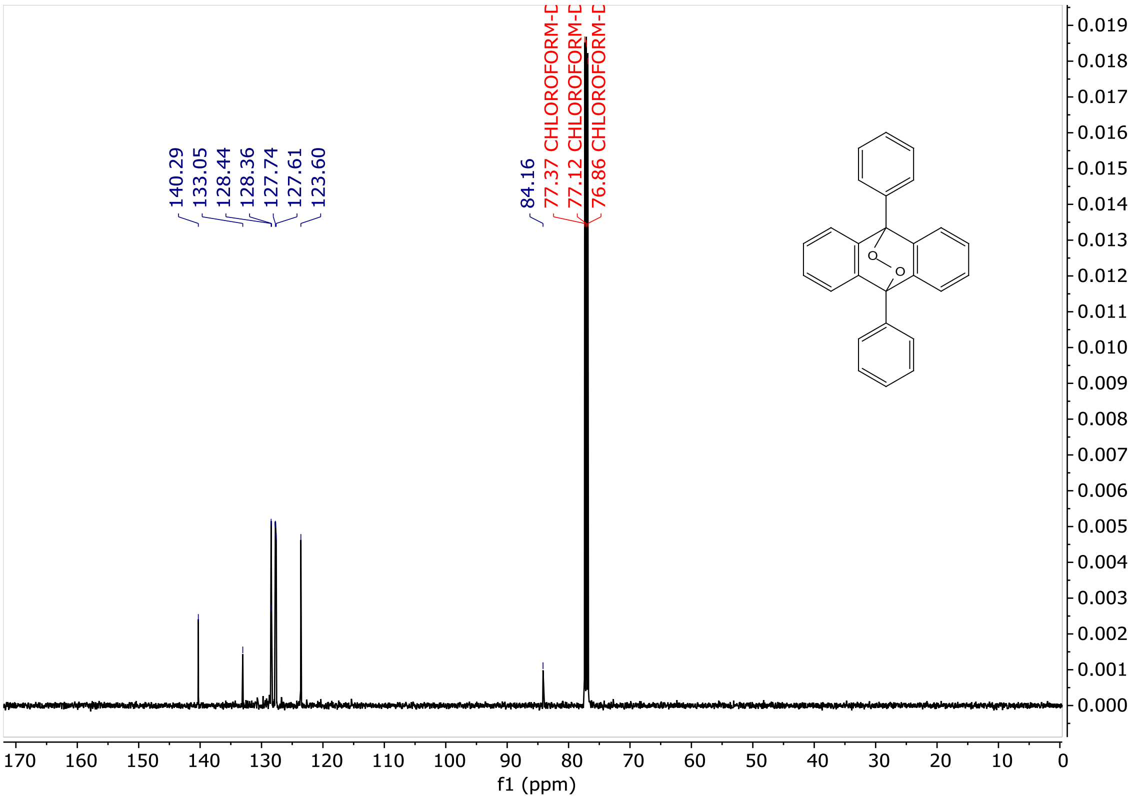

Figure S11: $^{13}C\{^{1}H\}$ NMR ($CDCl_3$, 101 MHz) spectrum of **3b**.

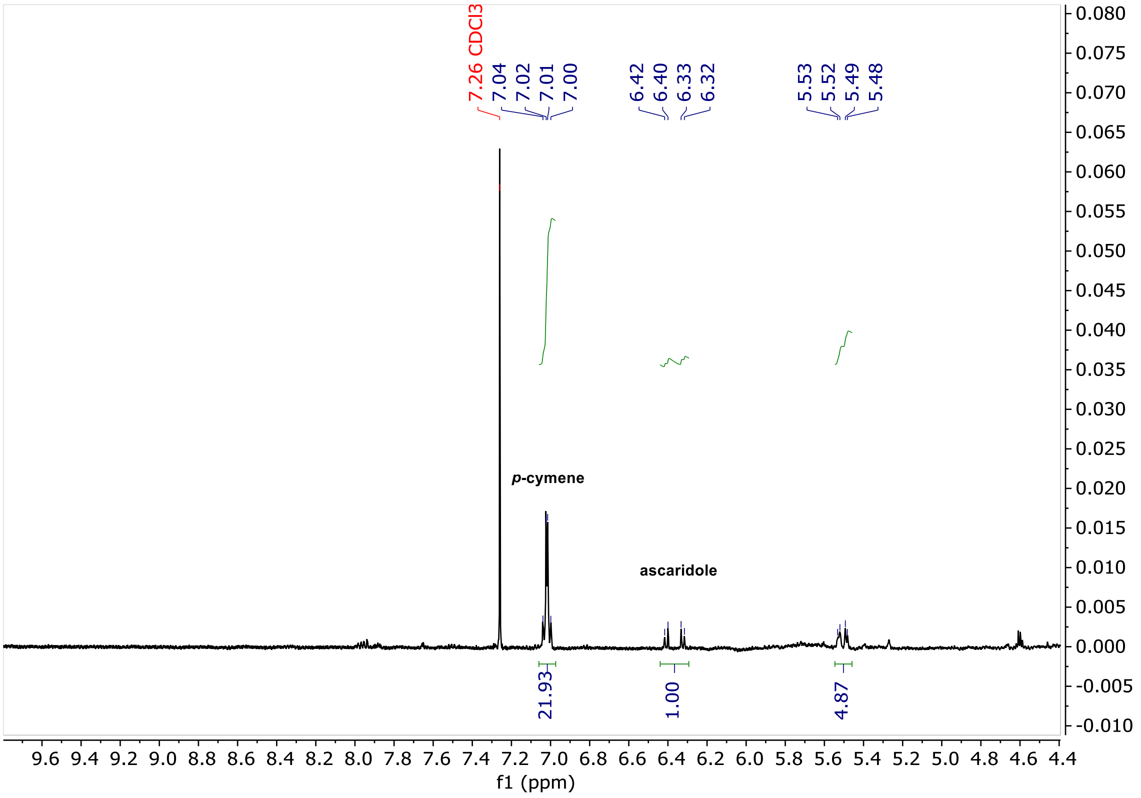

Figure S12: ^{1}H NMR ($CDCl_3$, 400 MHz) spectrum of crude a-terpinene irradiated in the presence of PQ for 30 min showing the p-cymene:ascaridole ratio.

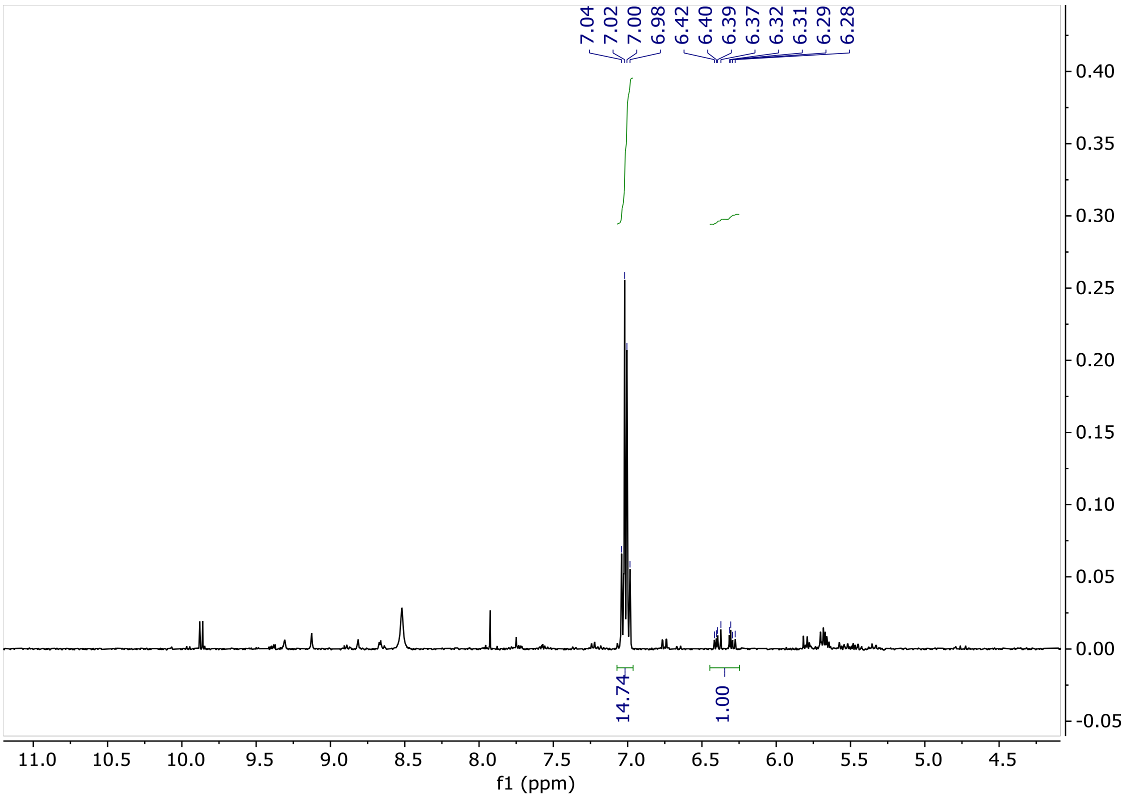

Figure S13: ^{1}H NMR ($CDCl_3$, 400 MHz) spectrum of crude a-terpinene irradiated in the presence of PQ for 4 min, showing further conversion into other oxidation products.

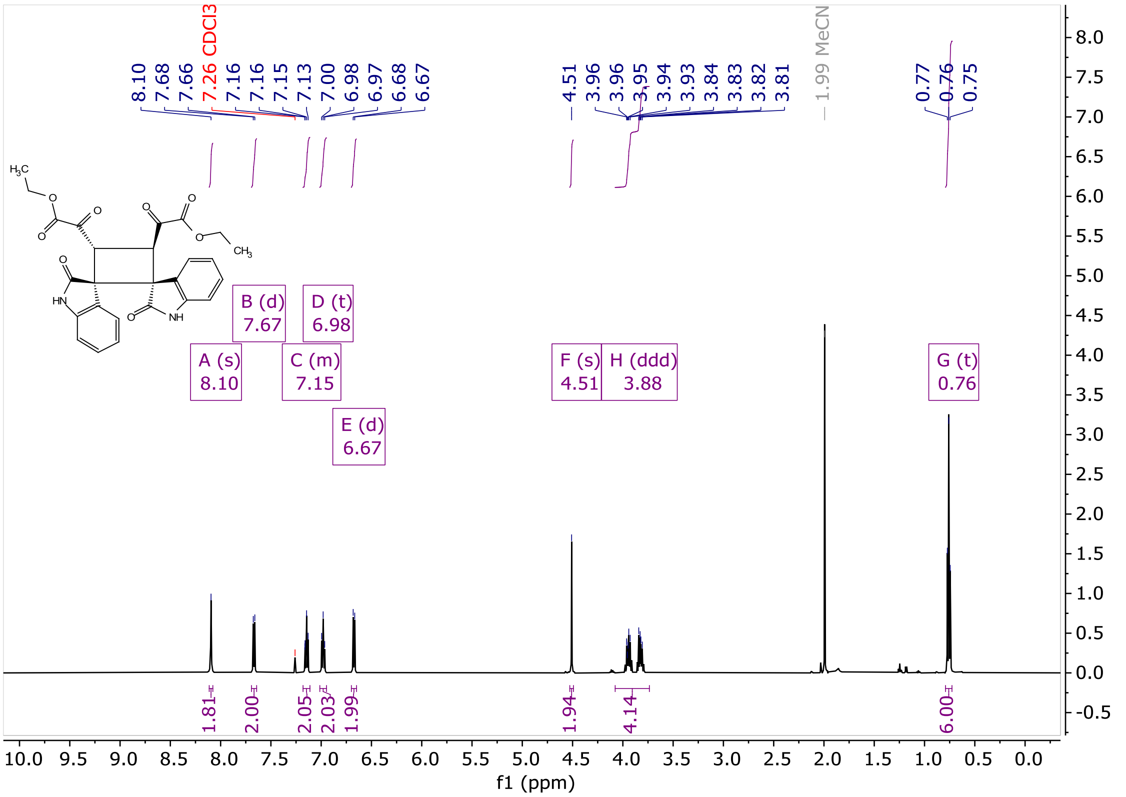

Figure S14: ^{1}H NMR ($CDCl_3$, 500 MHz) spectrum of **5b**.

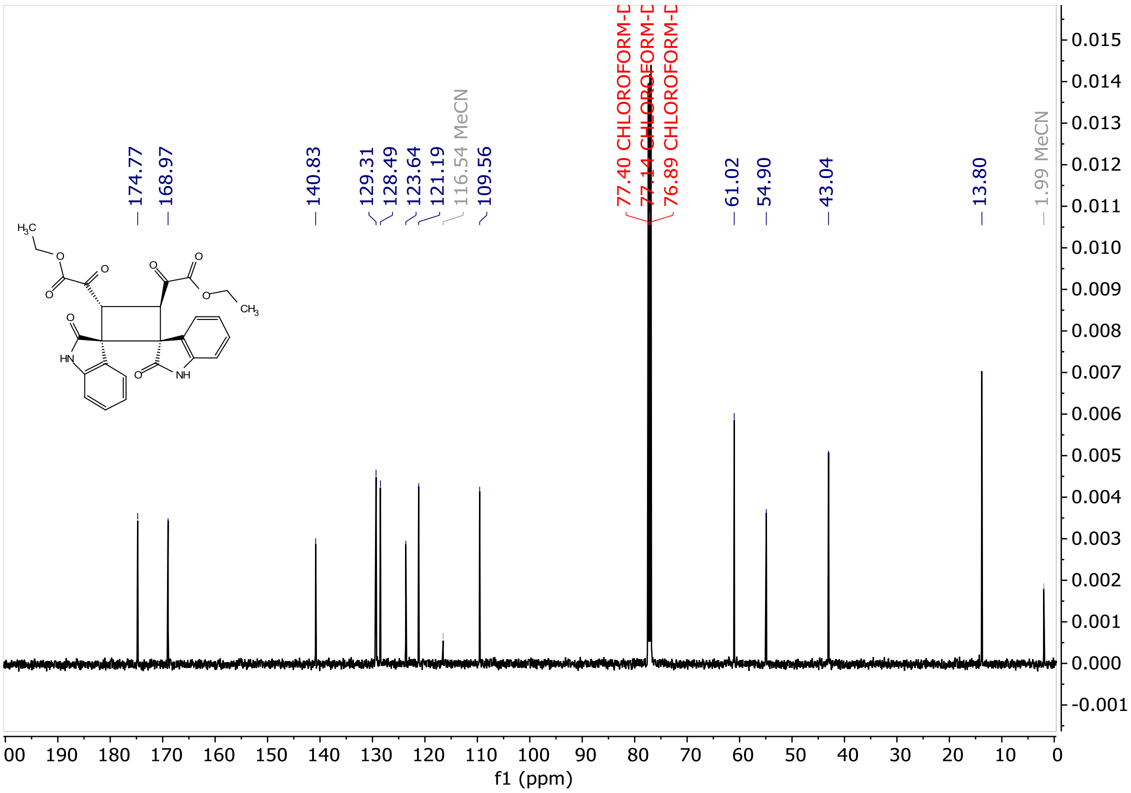

Figure S15: $^{13}C\{^{1}H\}$ NMR ($CDCl_3$, 126 MHz) spectrum of **5b**.

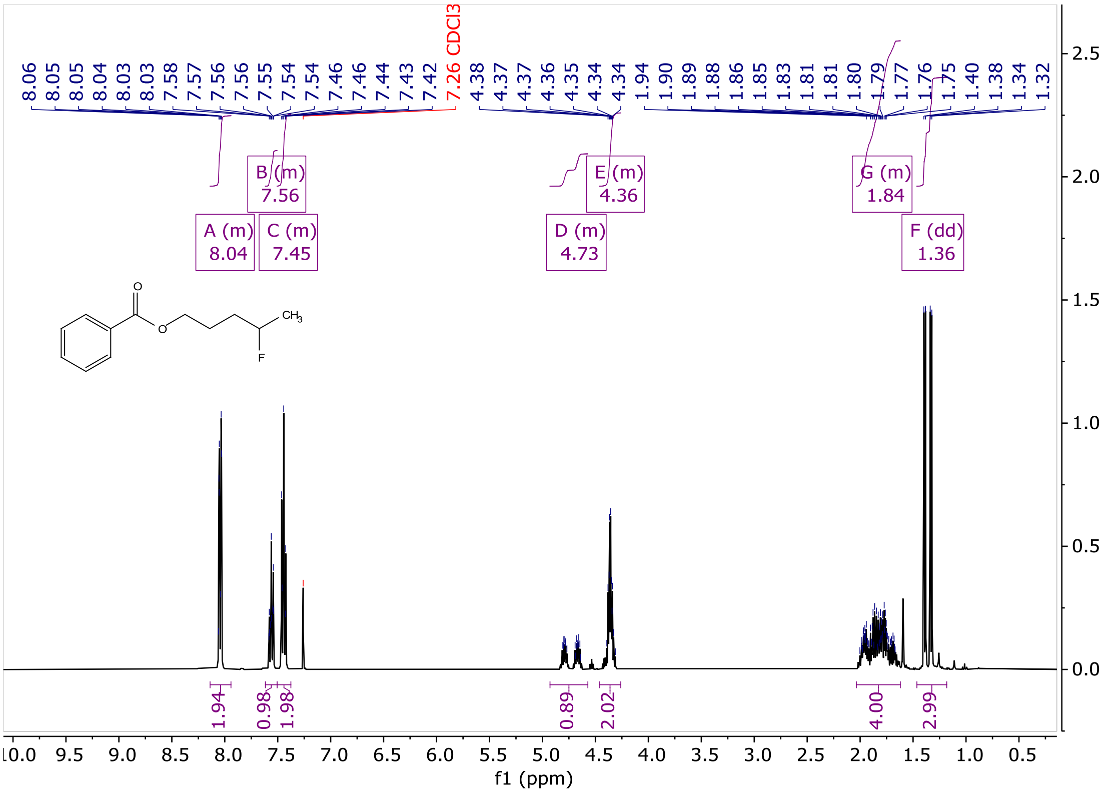

Figure S16: ^{1}H NMR ($CDCl_3$, 400 MHz) spectrum of **6b**.

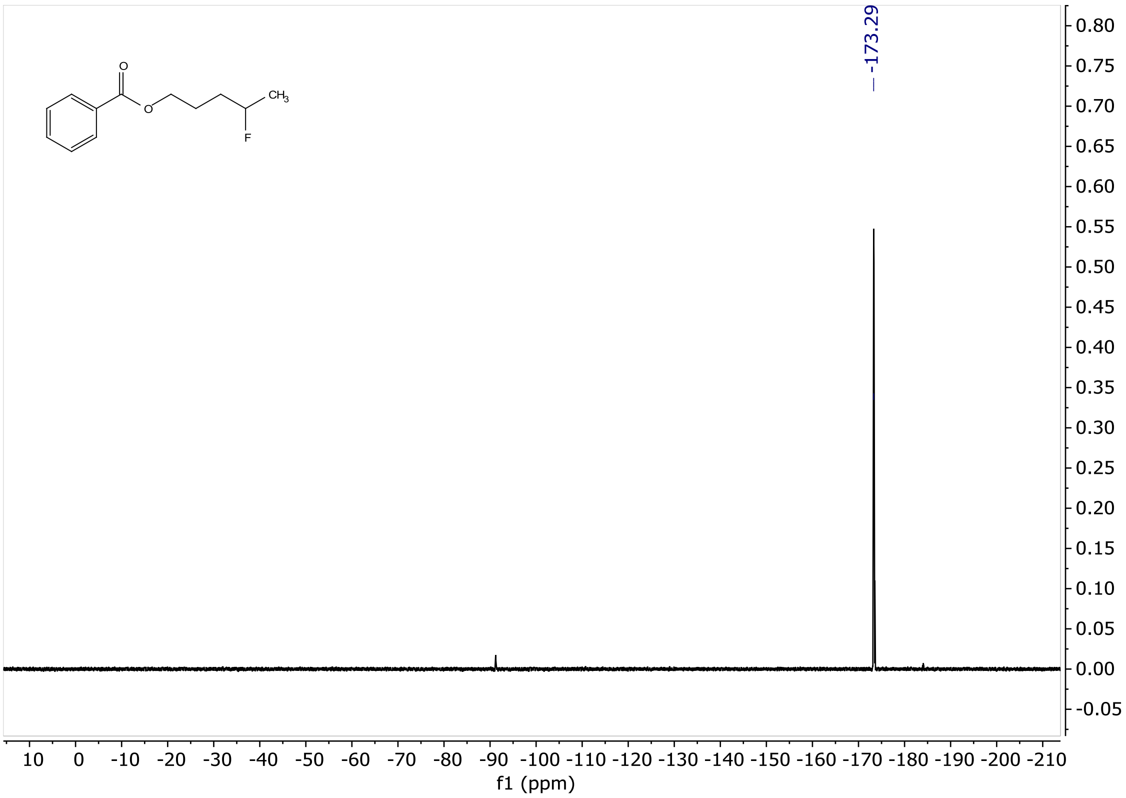

Figure S17: ^{19}F NMR ($CDCl_3$, 376 MHz) spectrum of **6b**.

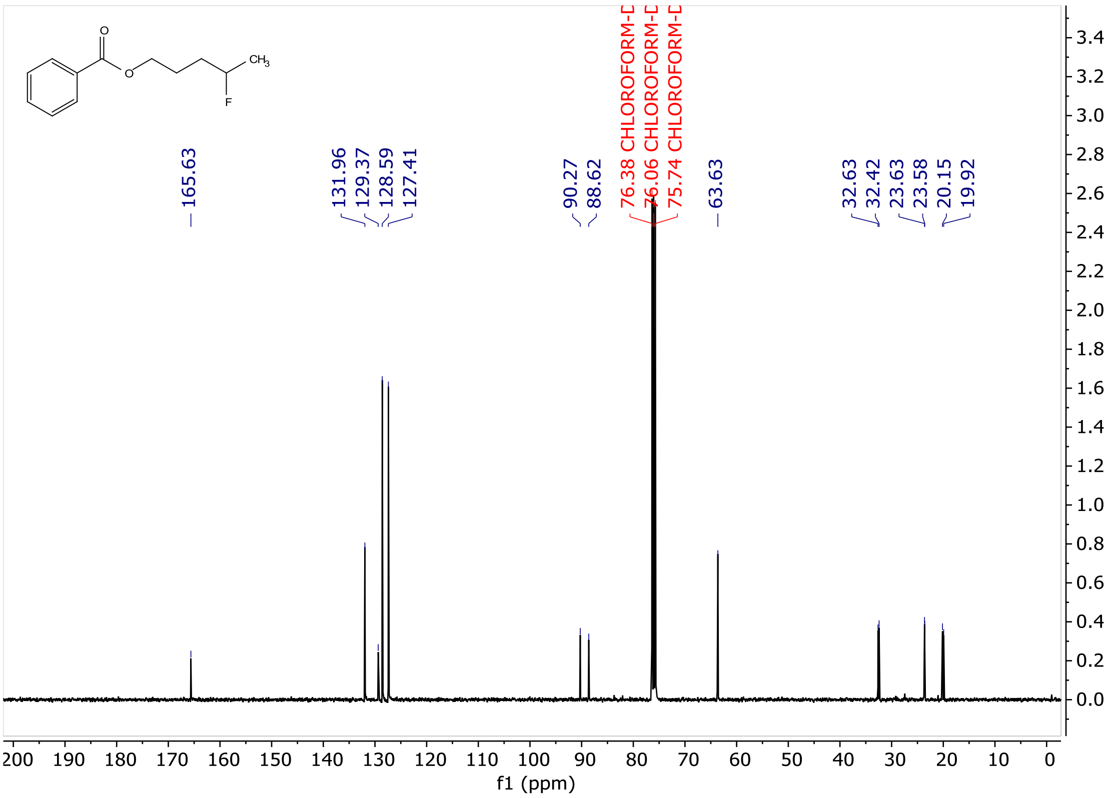

Figure S18: $^{13}C\{^{1}H\}$ NMR ($CDCl_3$, 101 MHz) spectrum of **6b**.

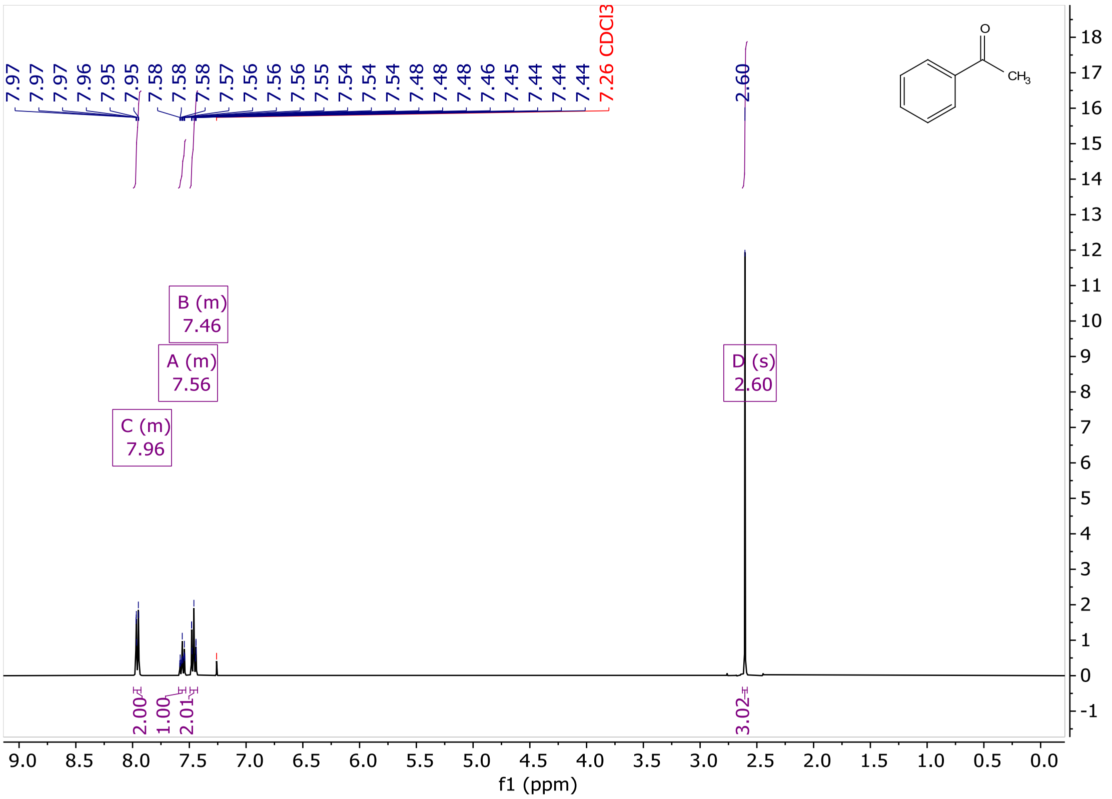

Figure S19: ^{1}H NMR ($CDCl_3$, 400 MHz) spectrum of **7b**.

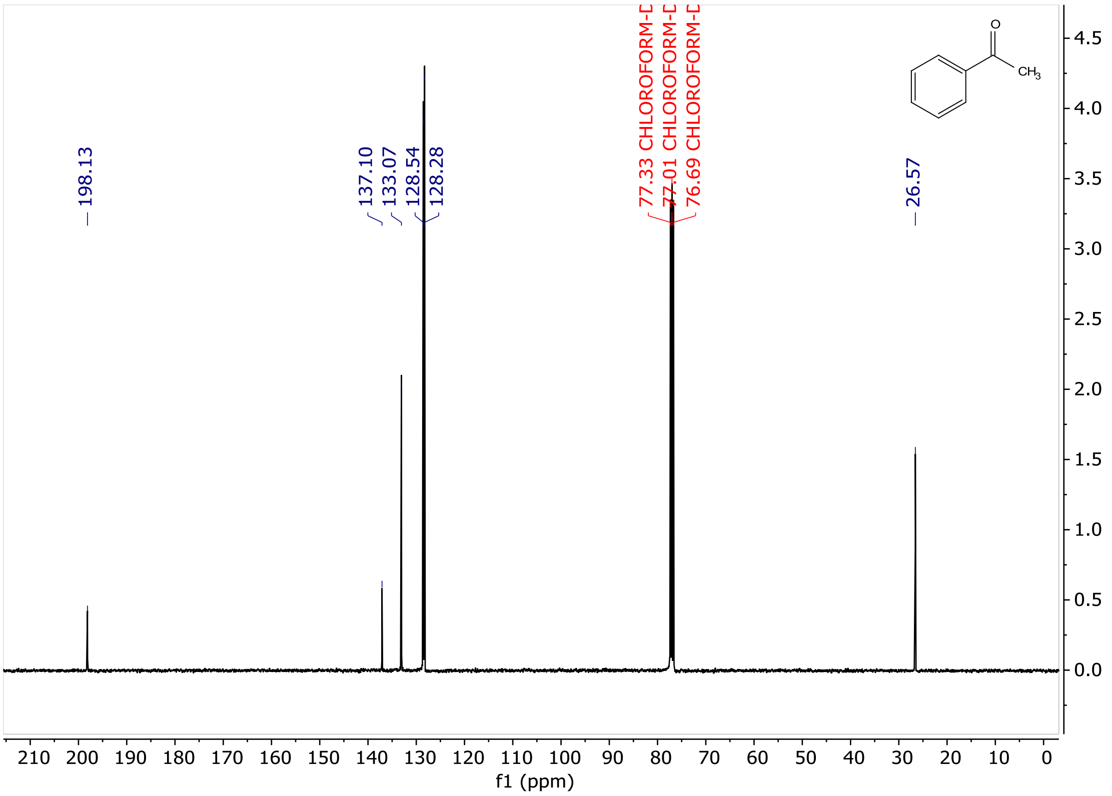

Figure S20: $^{13}C\{^{1}H\}$ NMR ($CDCl_3$, 101 MHz) spectrum of **7b**.

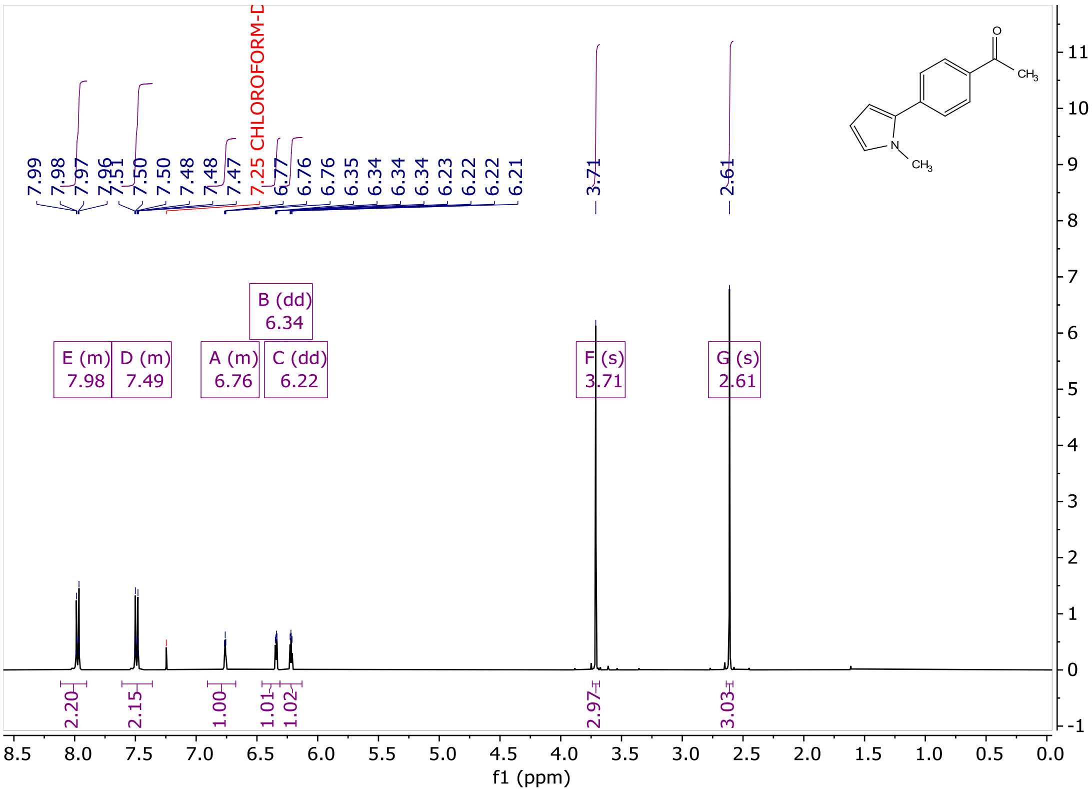

Figure S21: ^{1}H NMR ($CDCl_3$, 400 MHz) spectrum of **8b**.

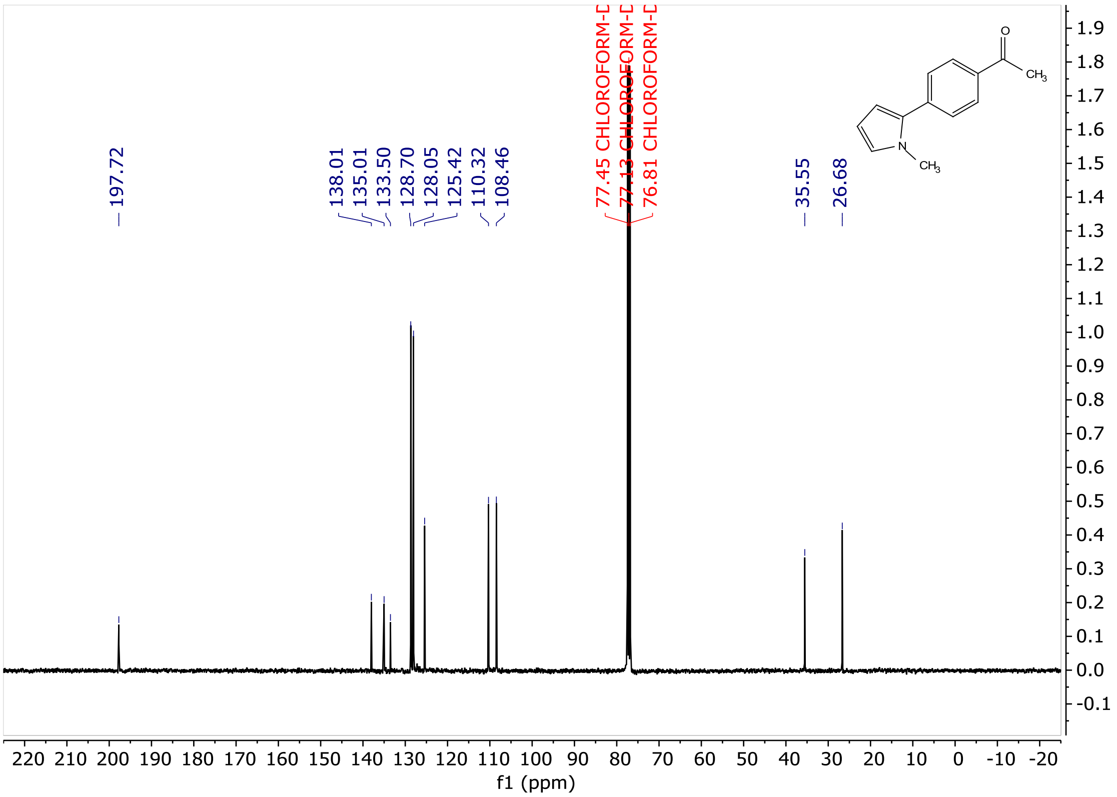

Figure S22: ^{13}C{^{1}H} NMR ($CDCl_3$, 101 MHz) spectrum of **8b**.

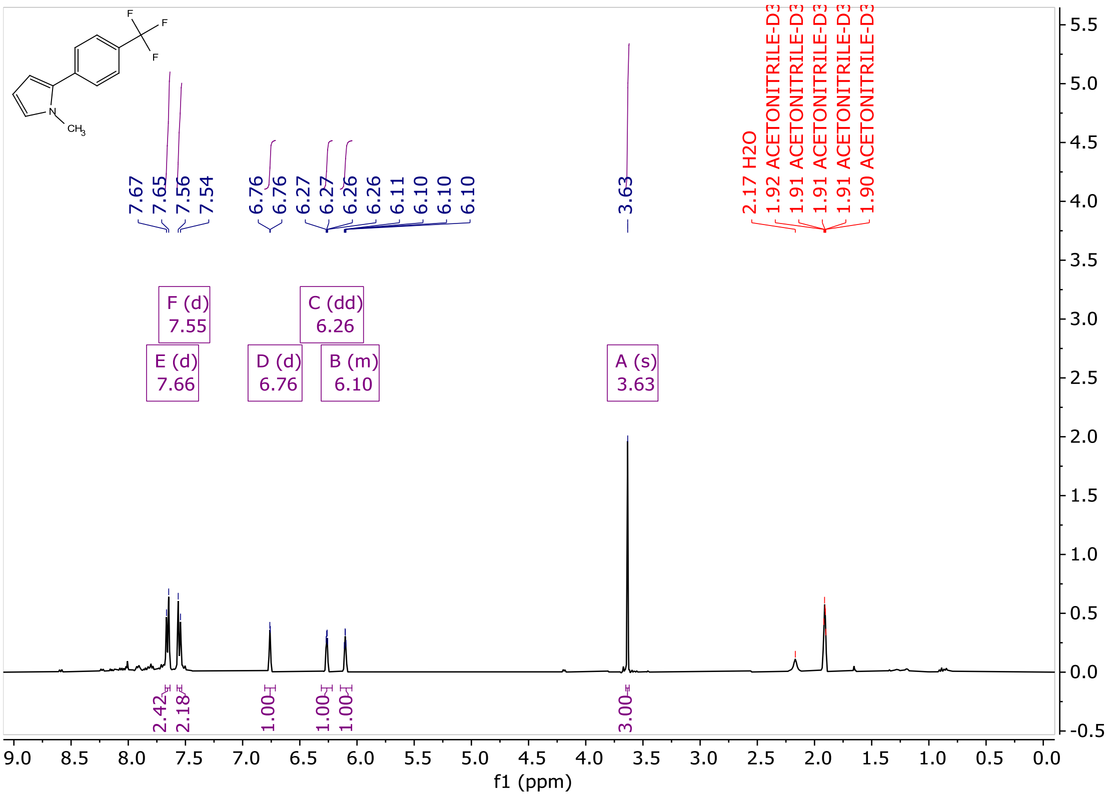

Figure S23: ^{1}H NMR (MeCN-d_3, 400 MHz) spectrum of **8c**.

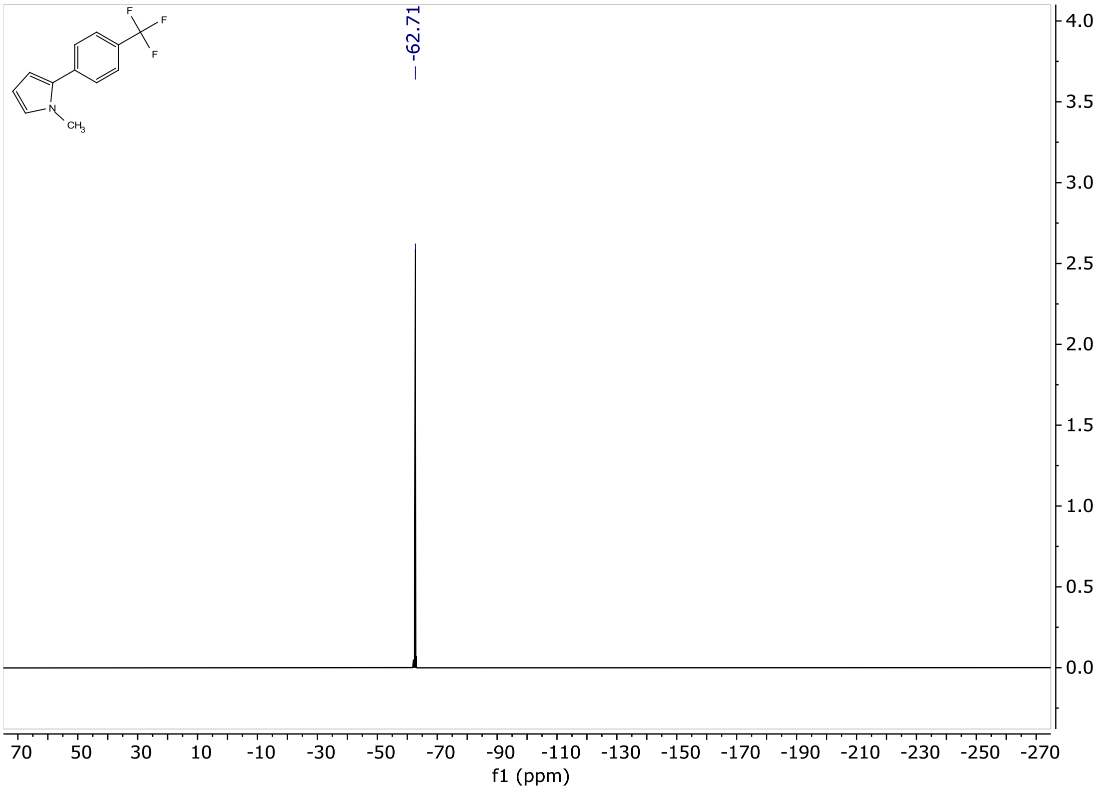

Figure S24: ^{19}F NMR (MeCN-d_3, 376 MHz) spectrum of **8c**.

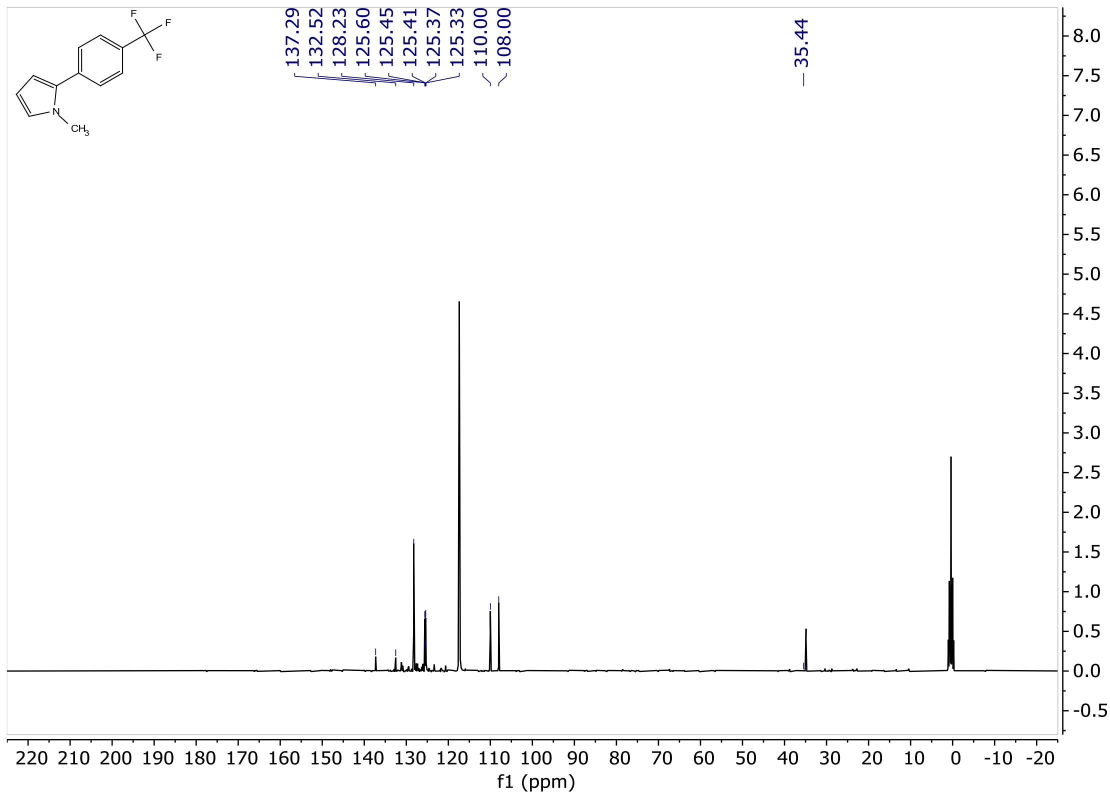

Figure S25: $^{13}C\{^1H\}$ NMR (MeCN-d_3, 101 MHz) spectrum of **8c**.

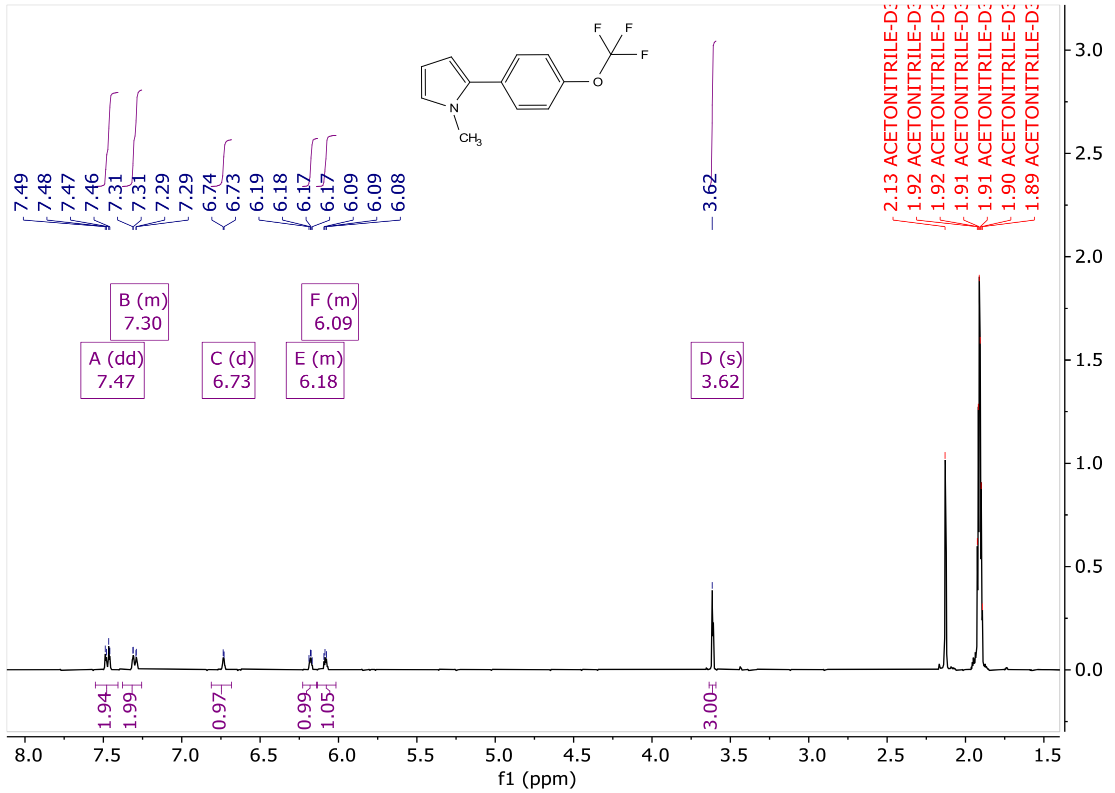

Figure S26: ^1H NMR (MeCN-d_3, 400 MHz) spectrum of **8d**.

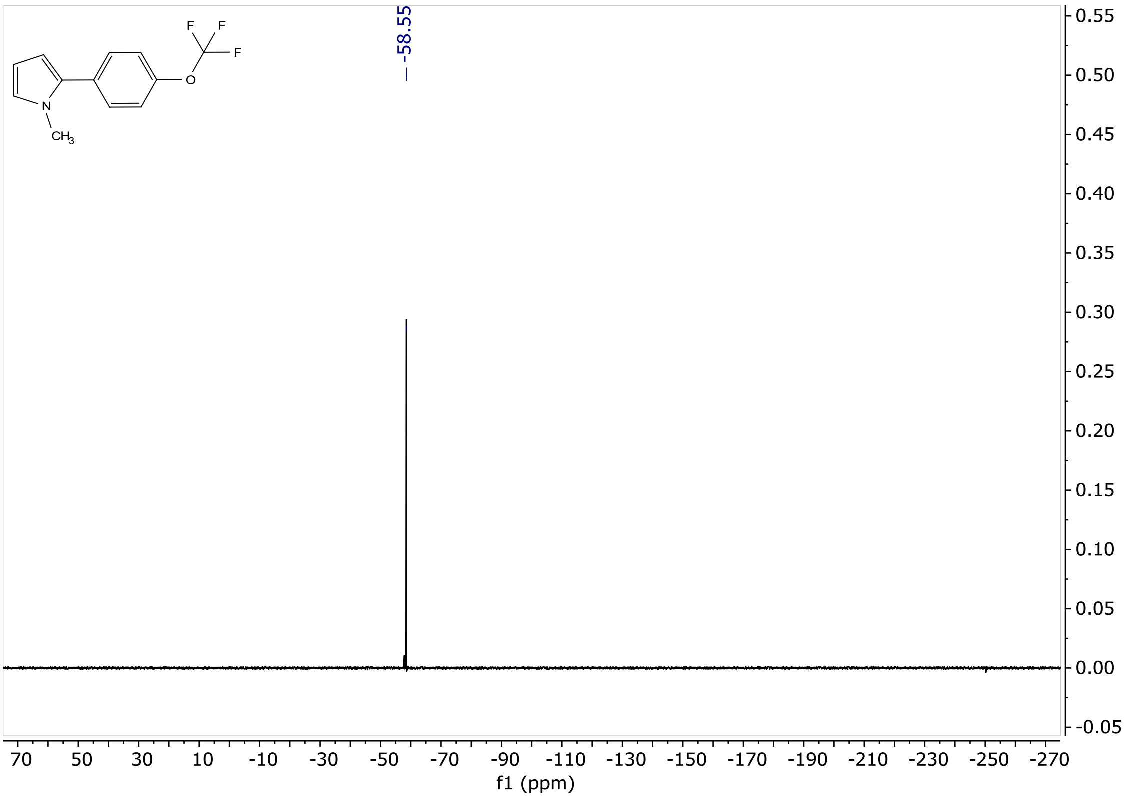

Figure S27: ^{19}F NMR (MeCN-d_3, 376 MHz) spectrum of **8d**.

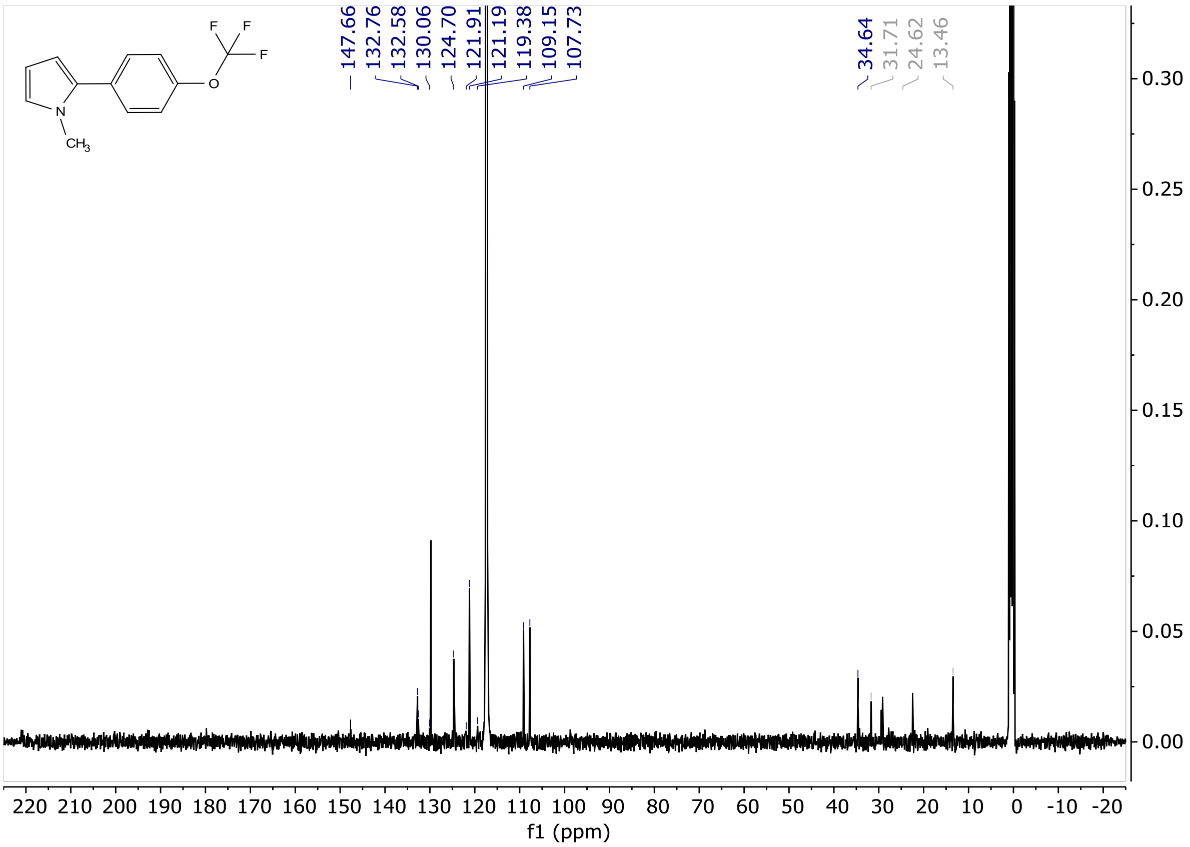

Figure S28: ^{13}C{^{1}H} NMR (MeCN-d_3, 101 MHz) spectrum of **8d**.

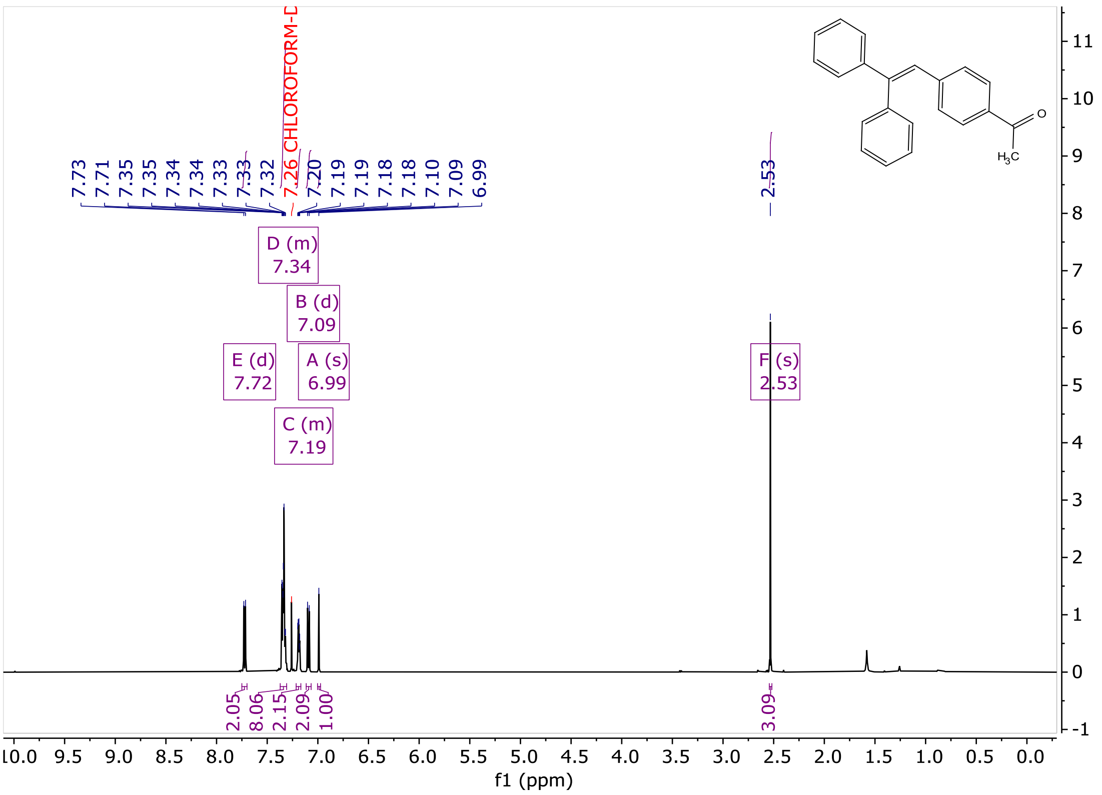

Figure S29: ^{1}H NMR ($CDCl_3$, 500 MHz) spectrum of **9b**.

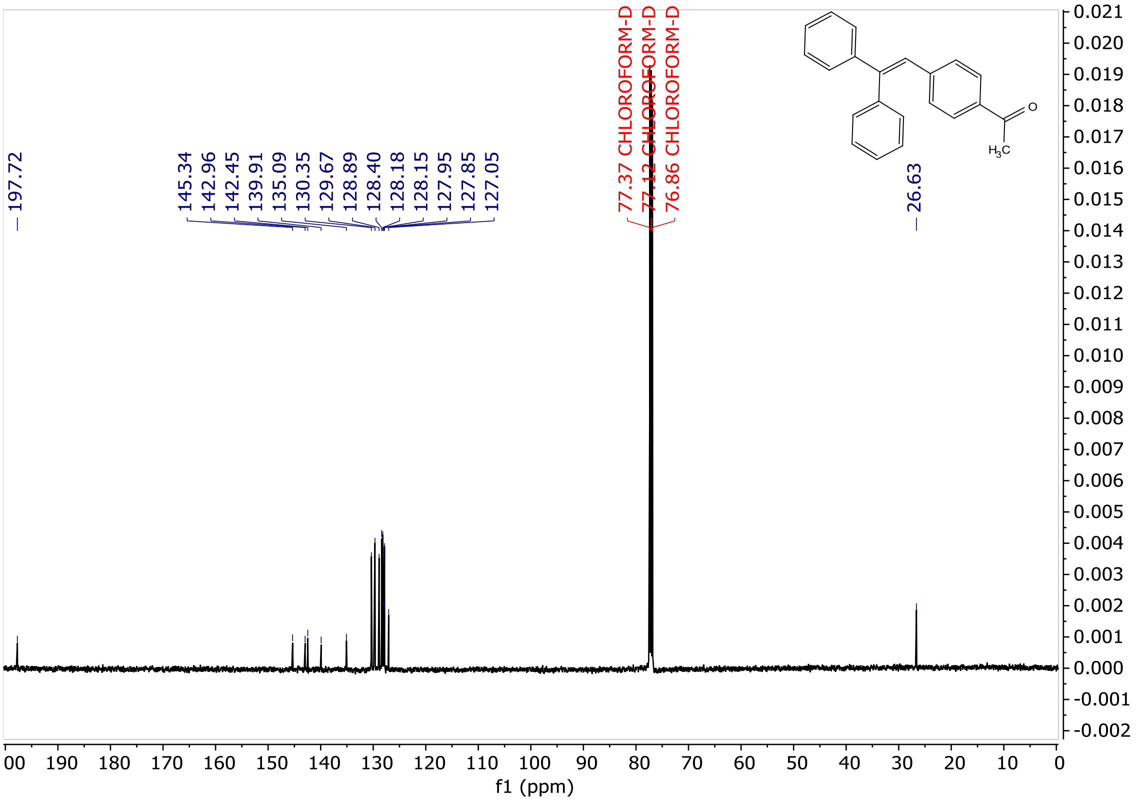

Figure S30: $^{13}C\{^1H\}$ NMR ($CDCl_3$, 126 MHz) spectrum of **9b**.

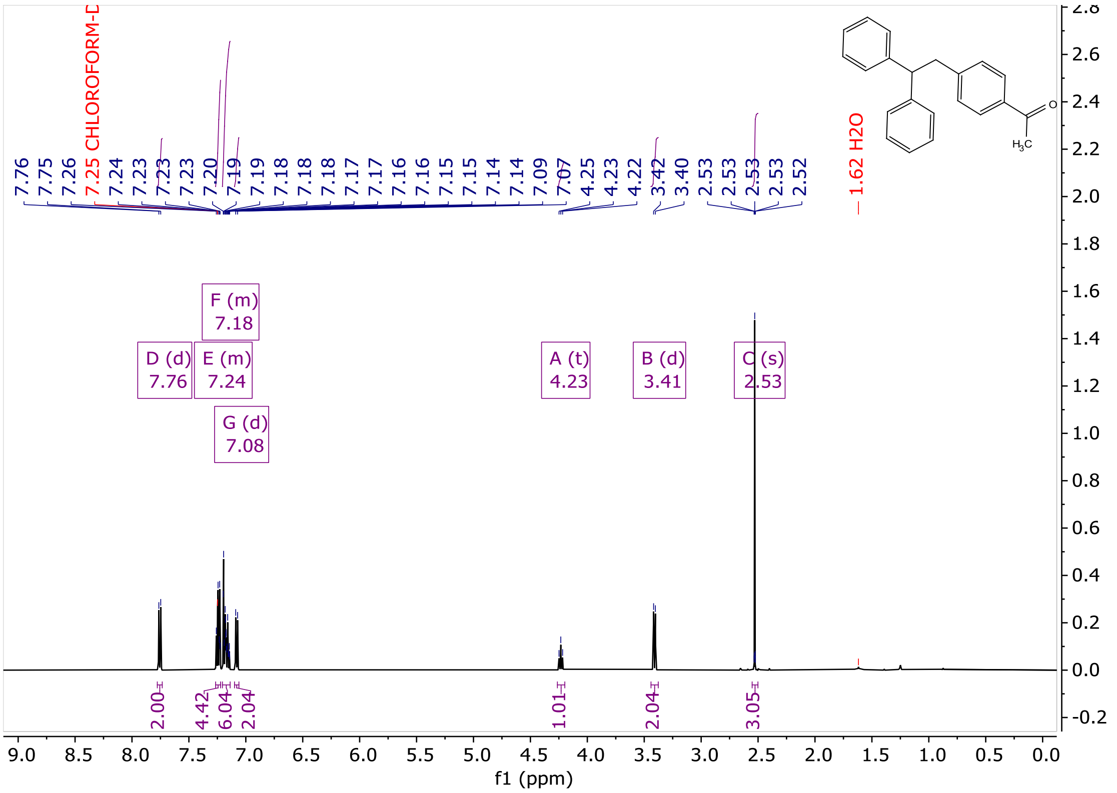

Figure S31: ^{1}H NMR ($CDCl_3$, 500 MHz) spectrum of **9c**.

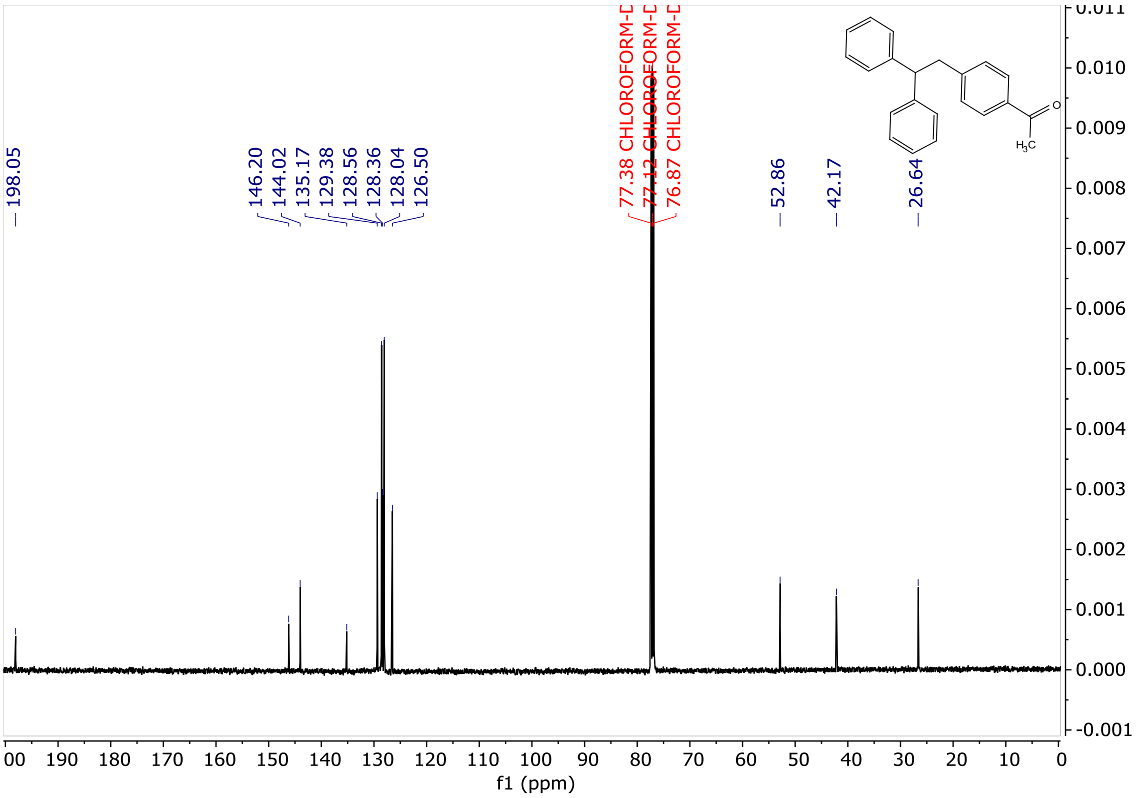

Figure S32: $^{13}C\{^{1}H\}$ NMR ($CDCl_3$, 126 MHz) spectrum of **9c**.

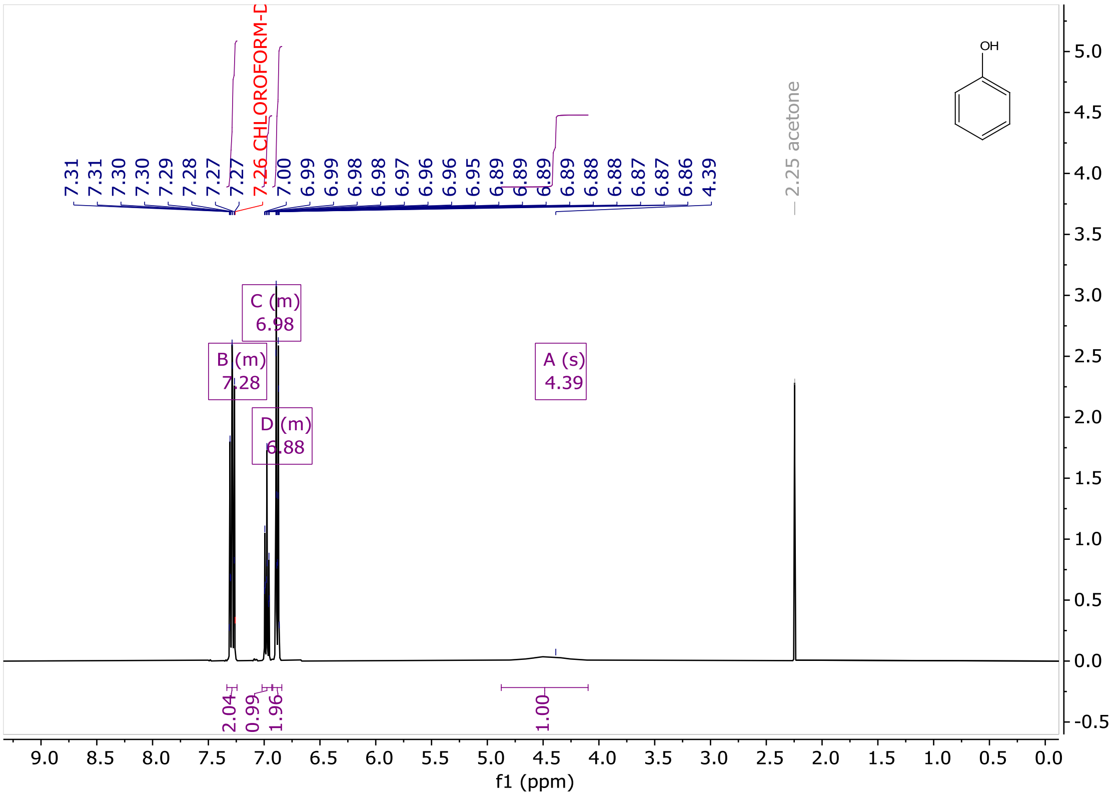

Figure S33: ^{1}H NMR ($CDCl_3$, 400 MHz) spectrum of **10b**.

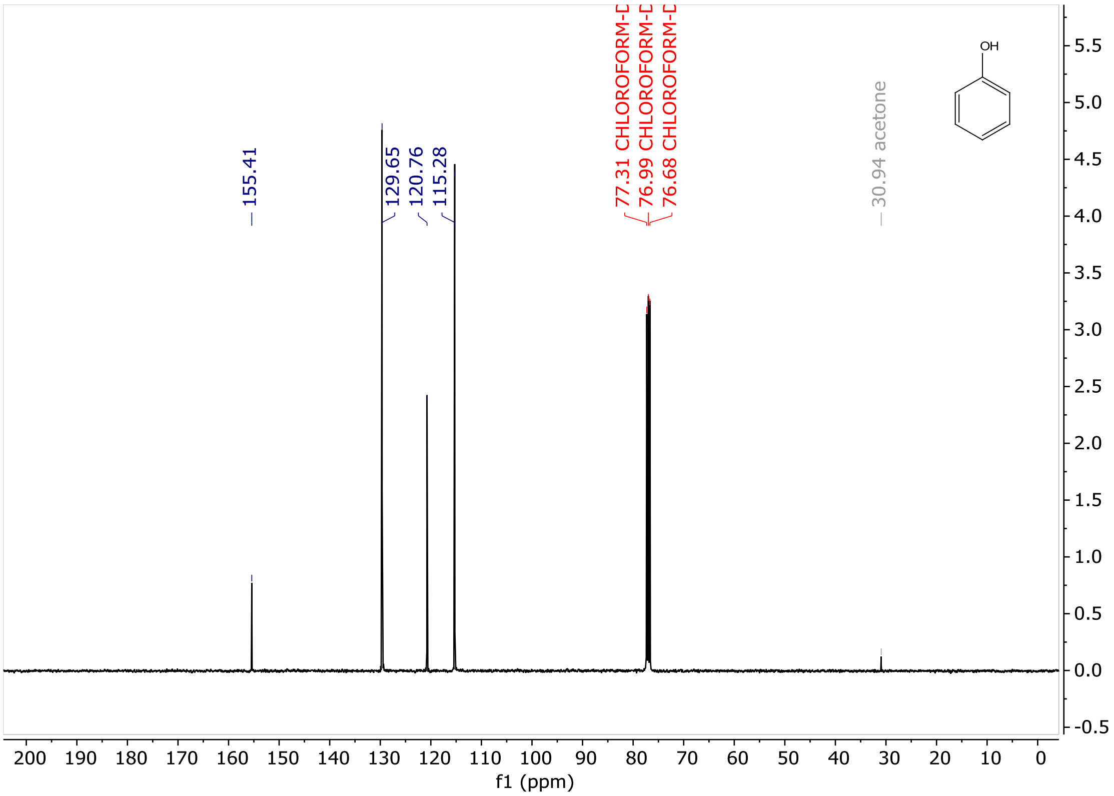

Figure S34: $^{13}C\{^{1}H\}$ NMR ($CDCl_3$, 101 MHz) spectrum of **10b**.

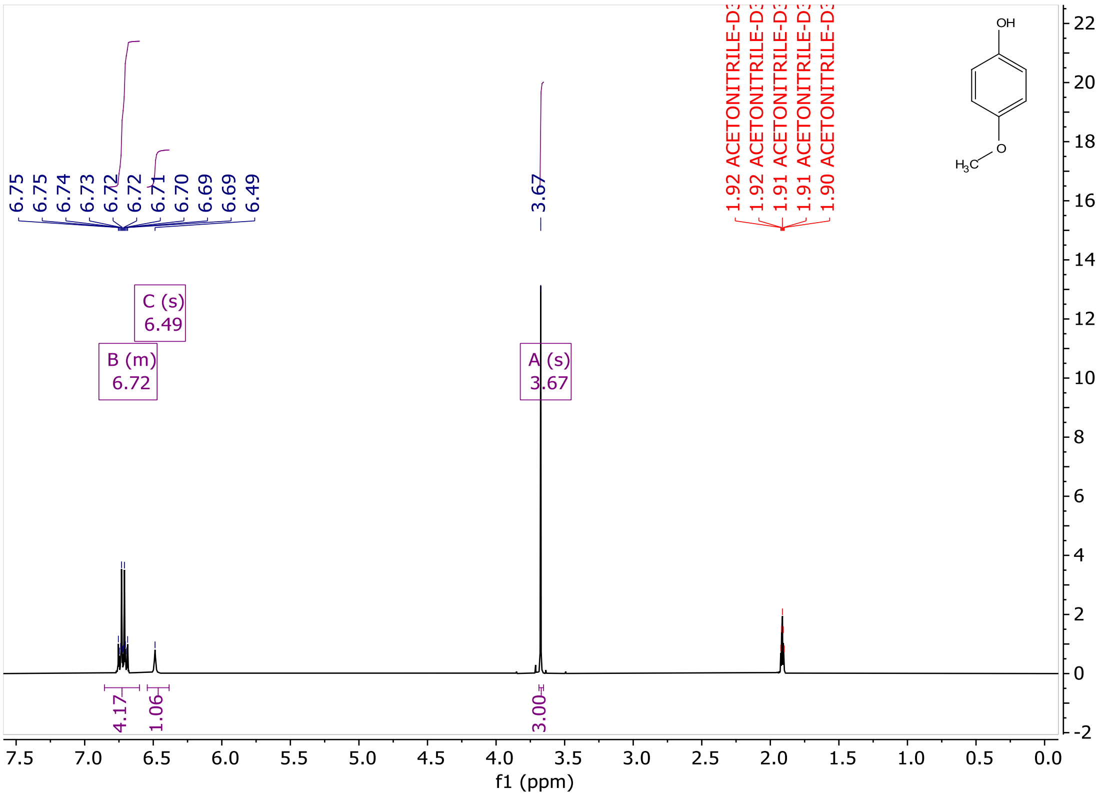

Figure S35: ^{1}H NMR (MeCN-d_3, 400 MHz) spectrum of **10c**.

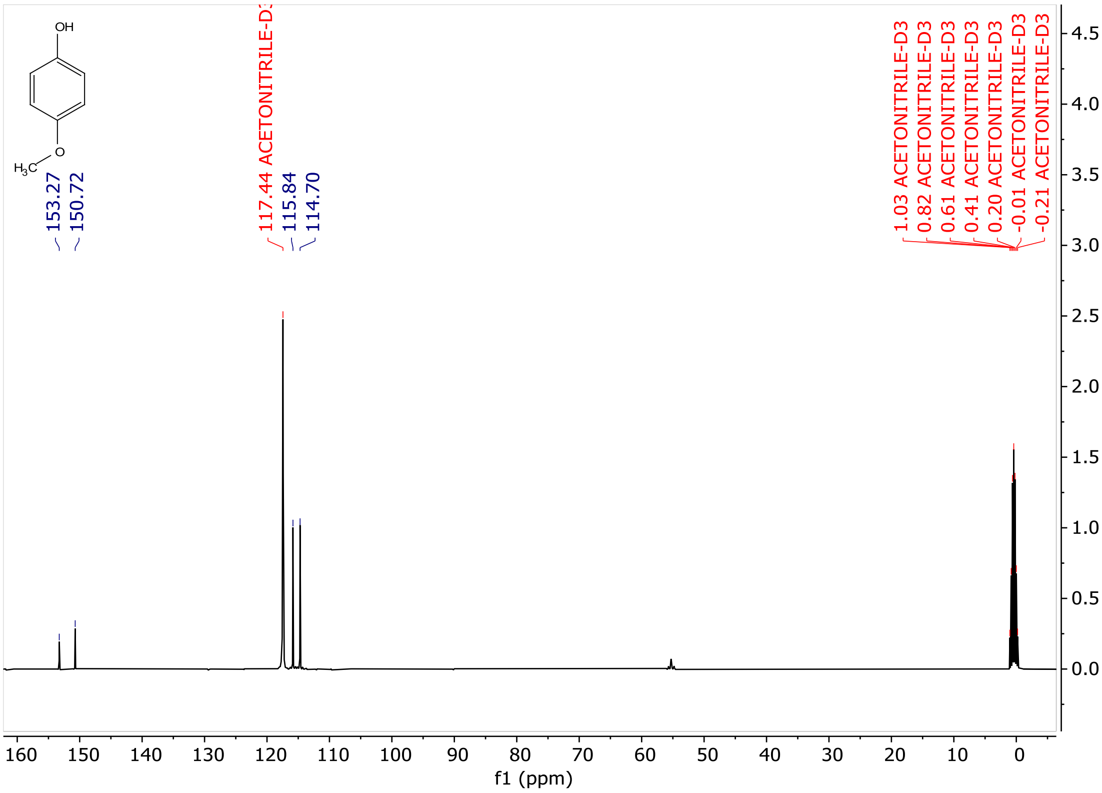

Figure S36: ^{13}C{^{1}H} NMR (MeCN-d_3, 101 MHz) spectrum of **10c**.

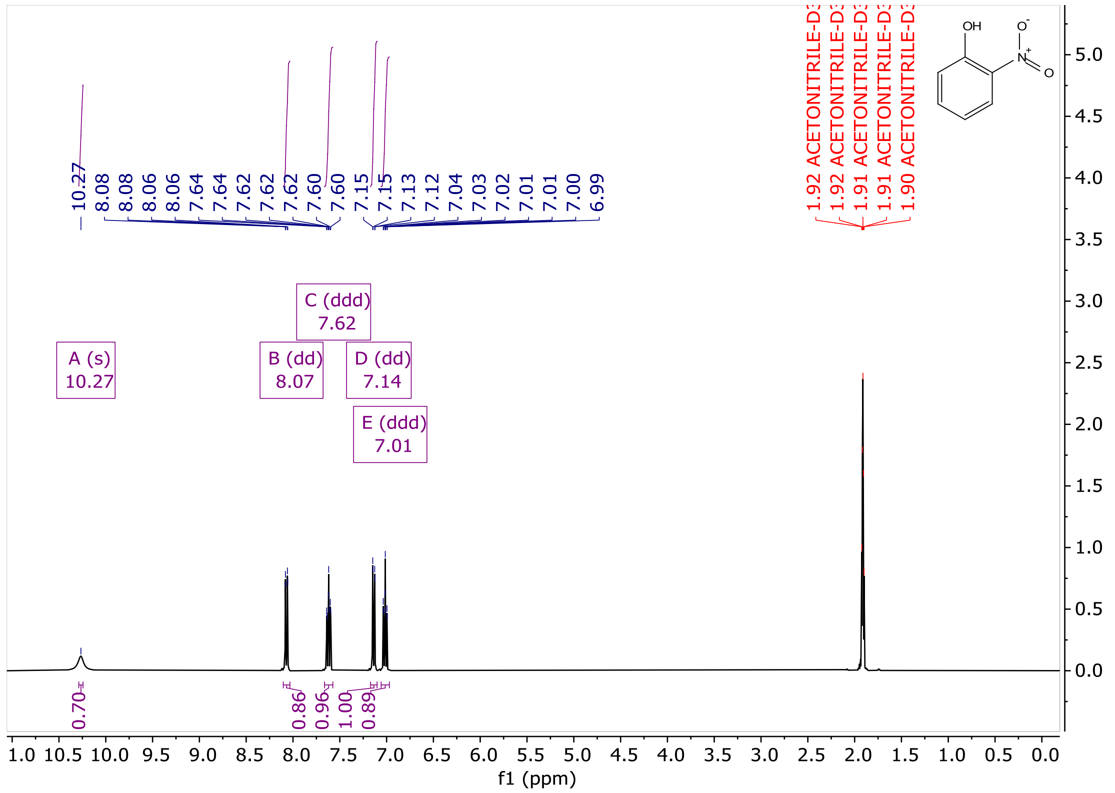

Figure S37: ^{1}H NMR (MeCN-d_3, 400 MHz) spectrum of **10d**.

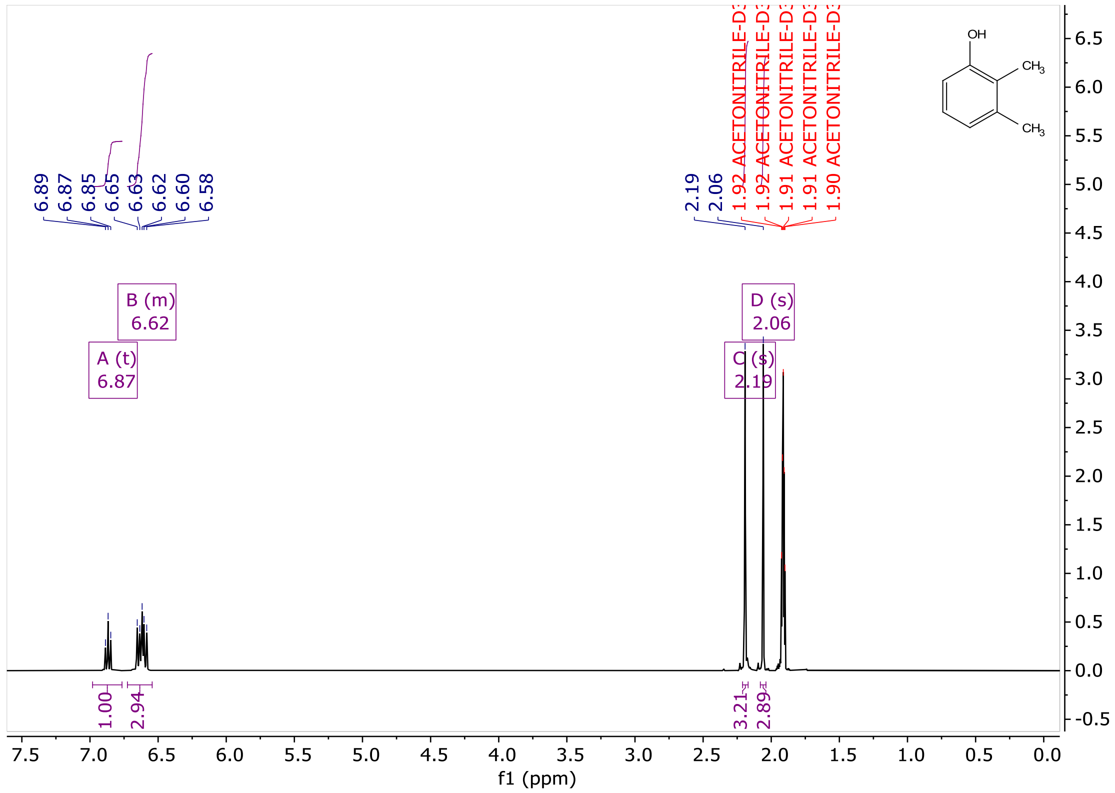

Figure S38: ^{1}H NMR (MeCN-d_3, 400 MHz) spectrum of **10e**.

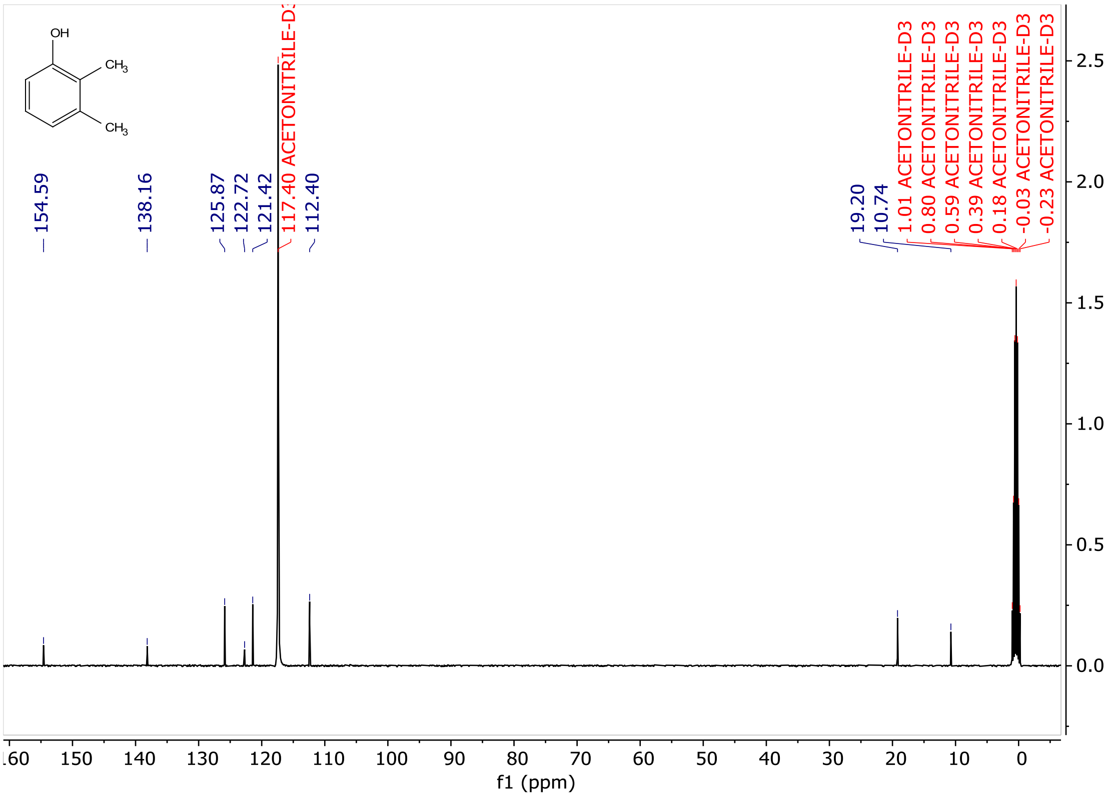

Figure S39: $^{13}C\{^1H\}$ NMR (MeCN-d_3, 101 MHz) spectrum of **10e**.

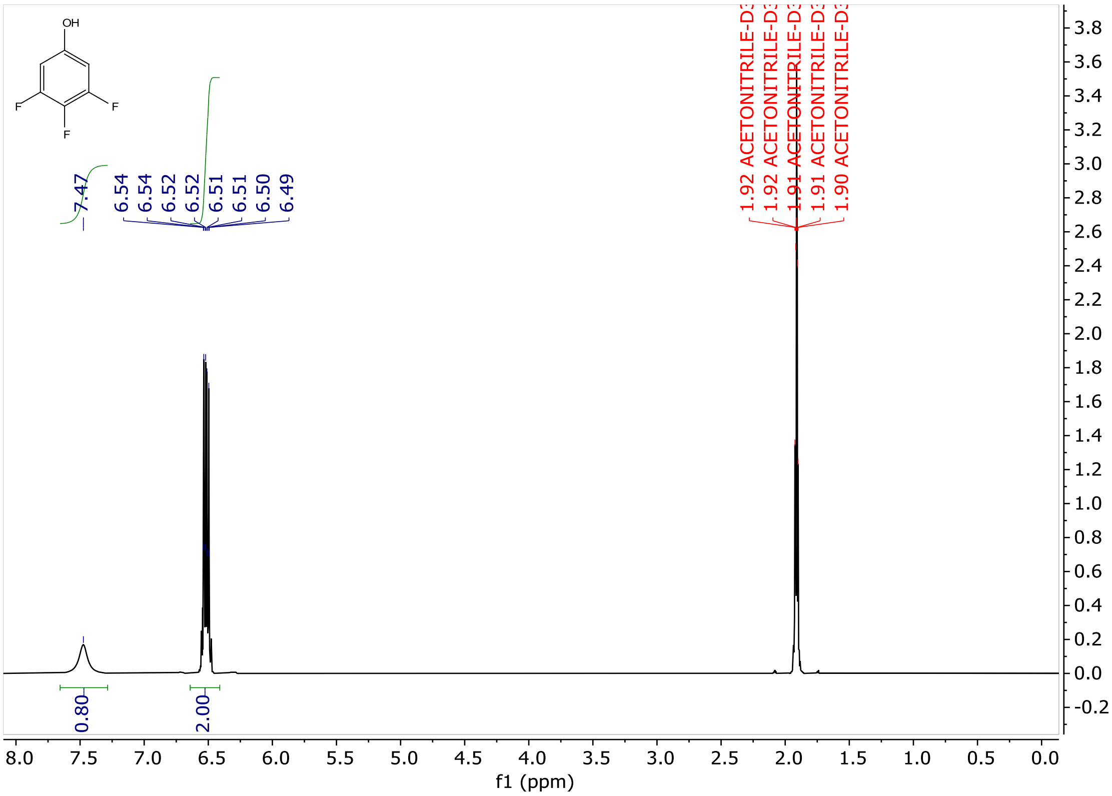

Figure S40: ^1H NMR (MeCN-d_3, 400 MHz) spectrum of **10f**.

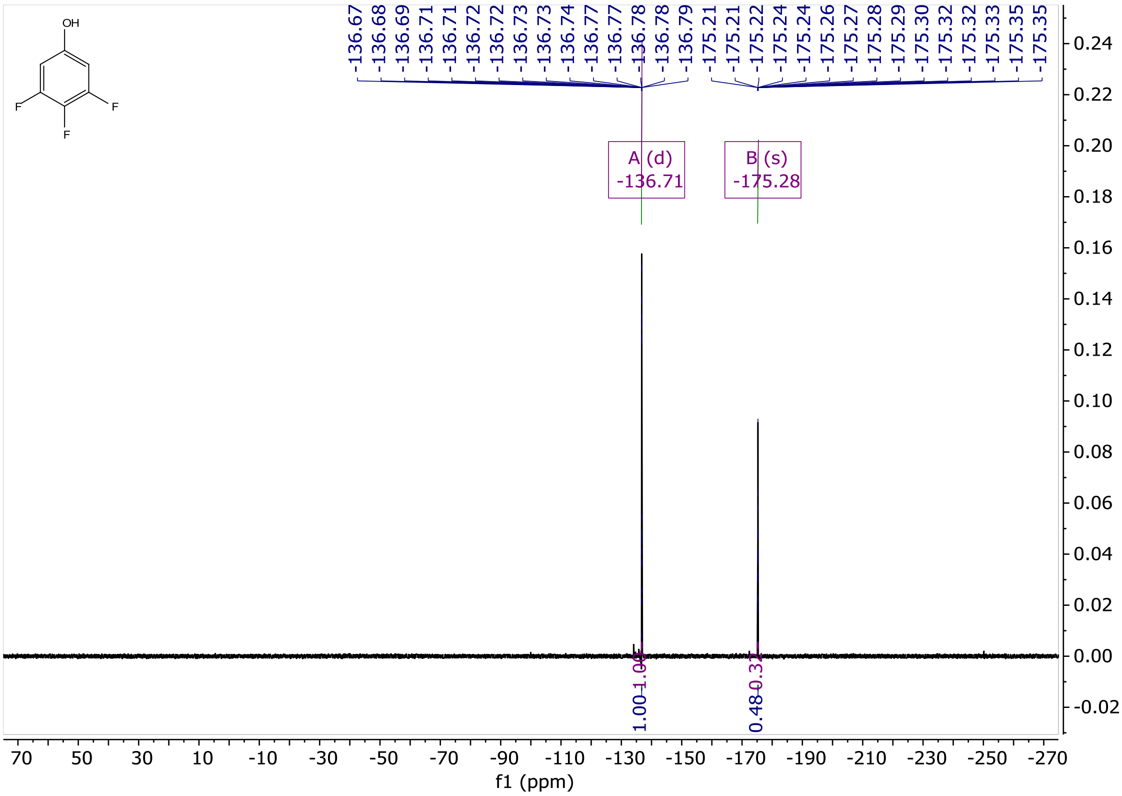

Figure S41: ^{19}F NMR (MeCN-d_3, 376 MHz) spectrum of **10f**.

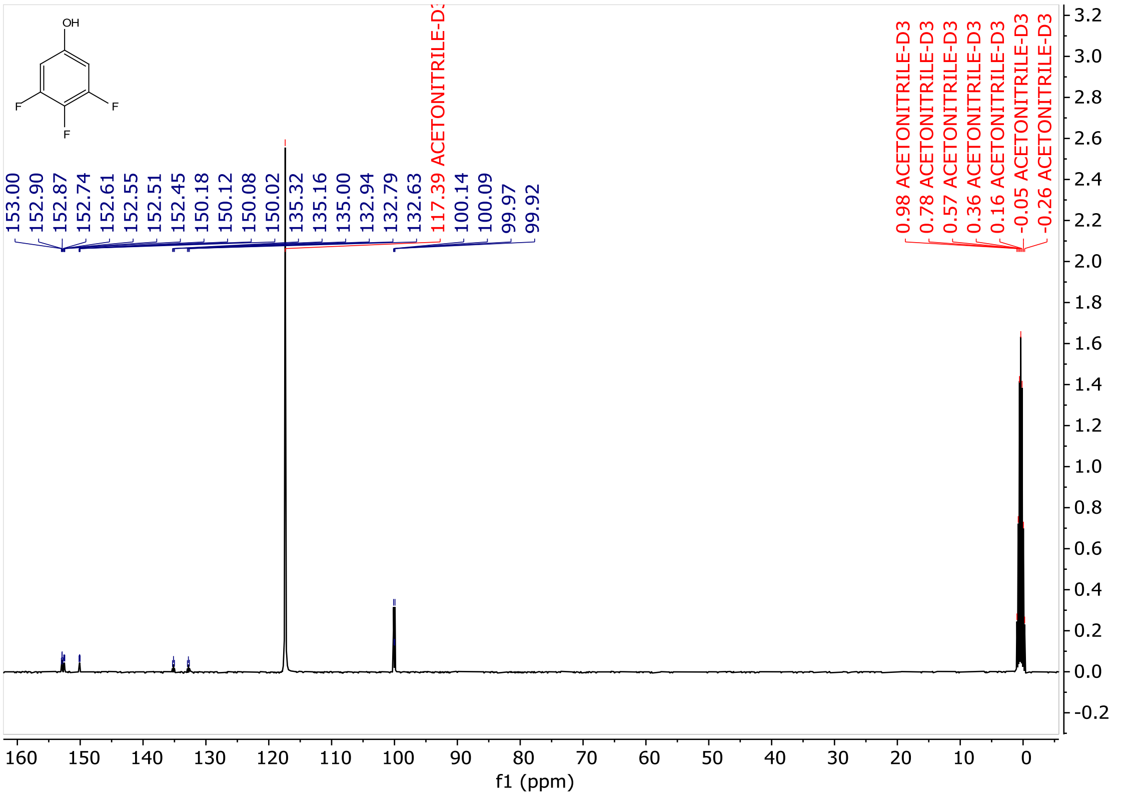

Figure S42: ^{13}C{^{1}H} NMR (MeCN-d_3, 101 MHz) spectrum of **10f**.

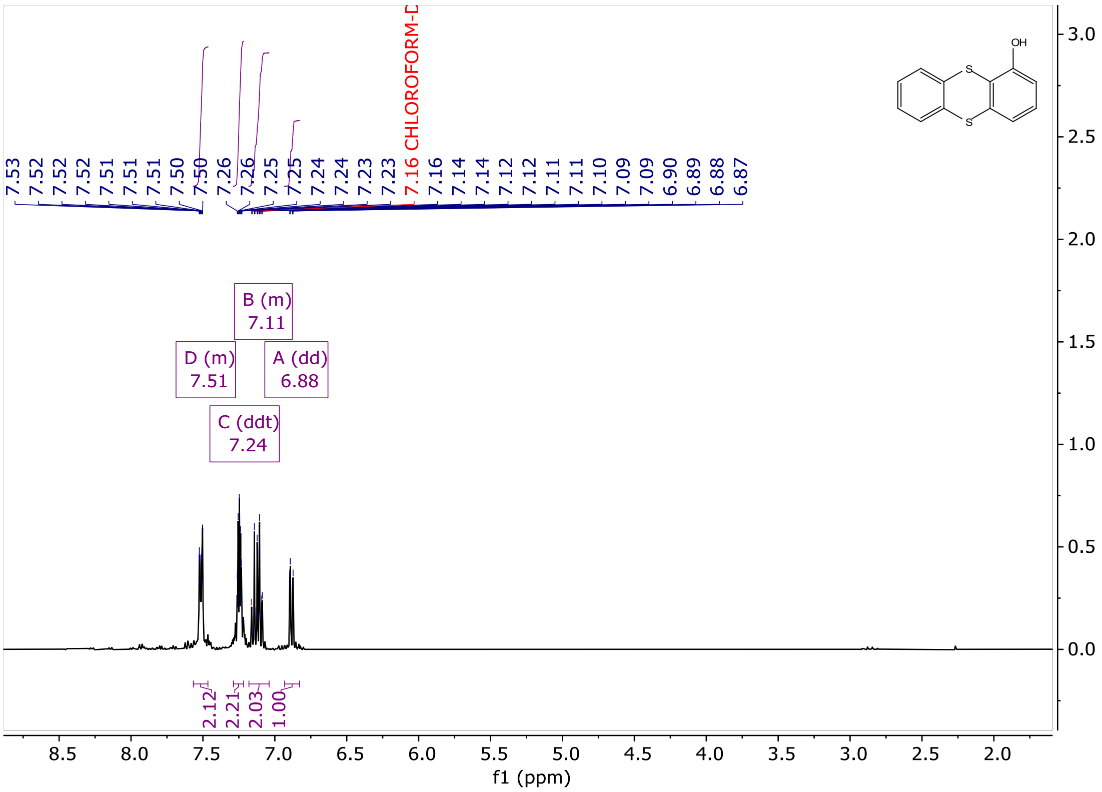

Figure S43: ^{1}H NMR ($CDCl_3$, 500 MHz) spectrum of **10g**.

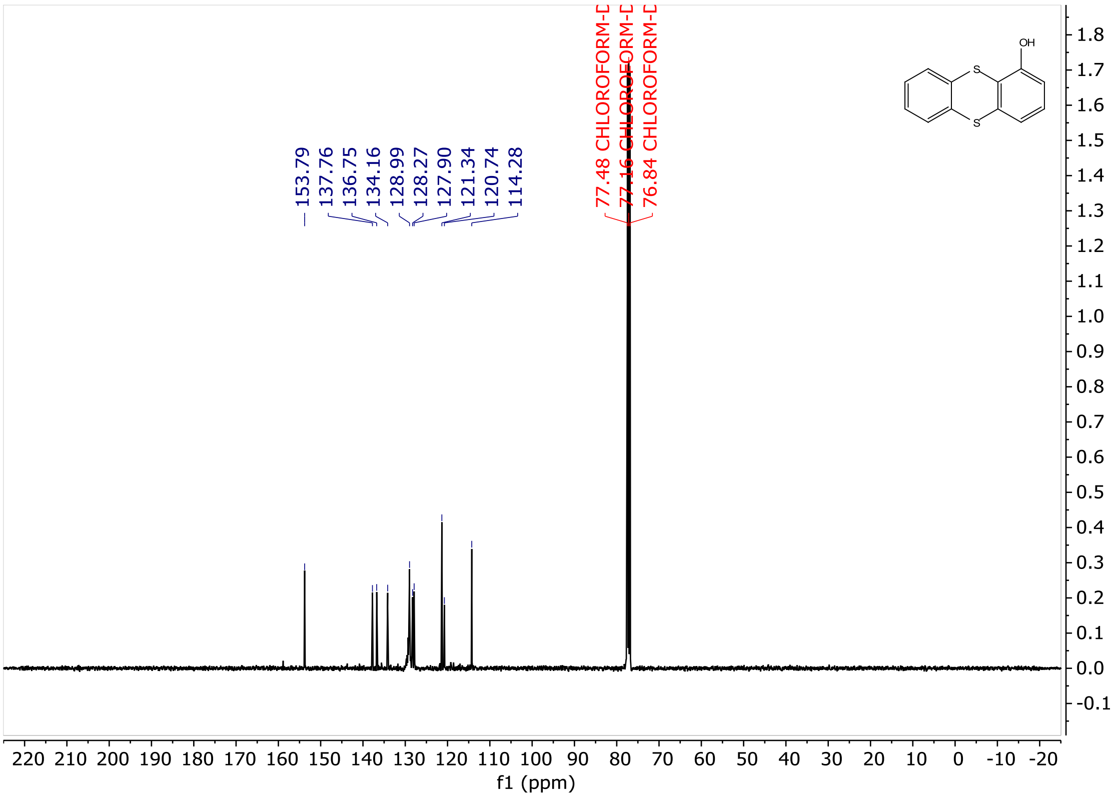

Figure S44: $^{13}C\{^{1}H\}$ NMR ($CDCl_3$, 101 MHz) spectrum of **10g**.

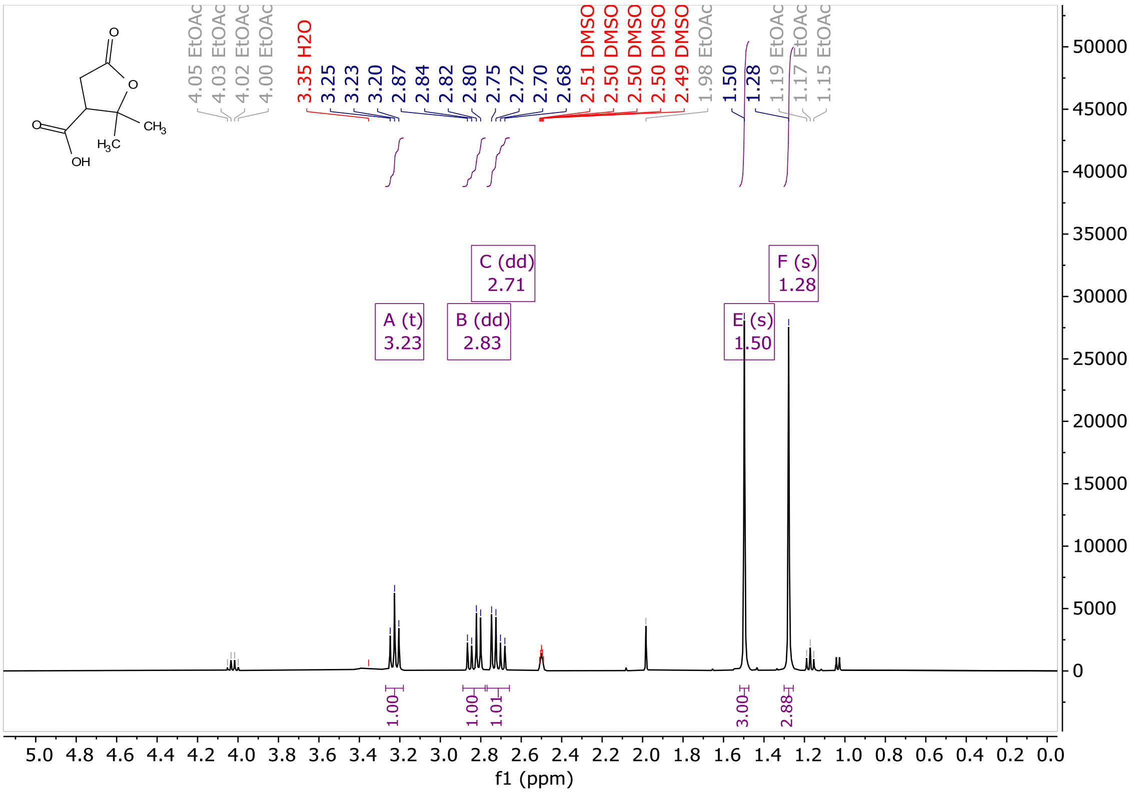

Figure S45: ^{1}H NMR (DMSO-d_6, 400 MHz) spectrum of terebic acid (**11b**)

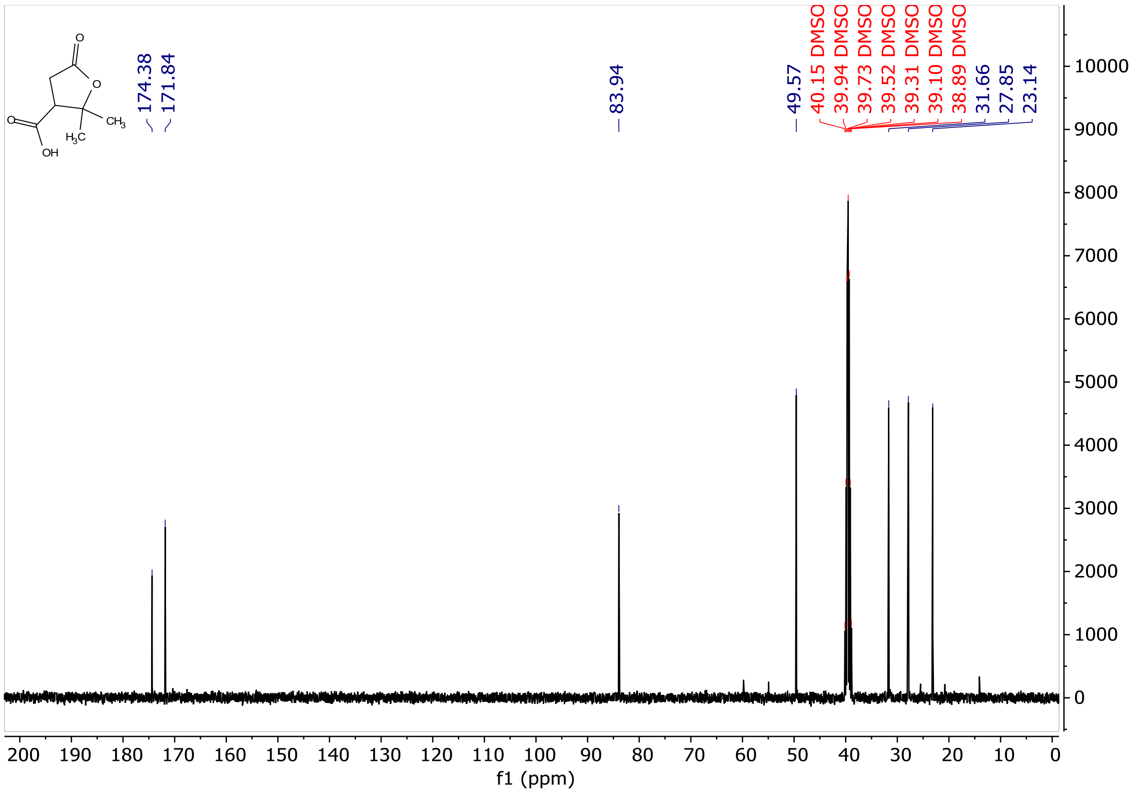

Figure S46: ^{13}C{^{1}H} NMR (DMSO-d_6, 101 MHz) spectrum of terebic acid (**11b**)

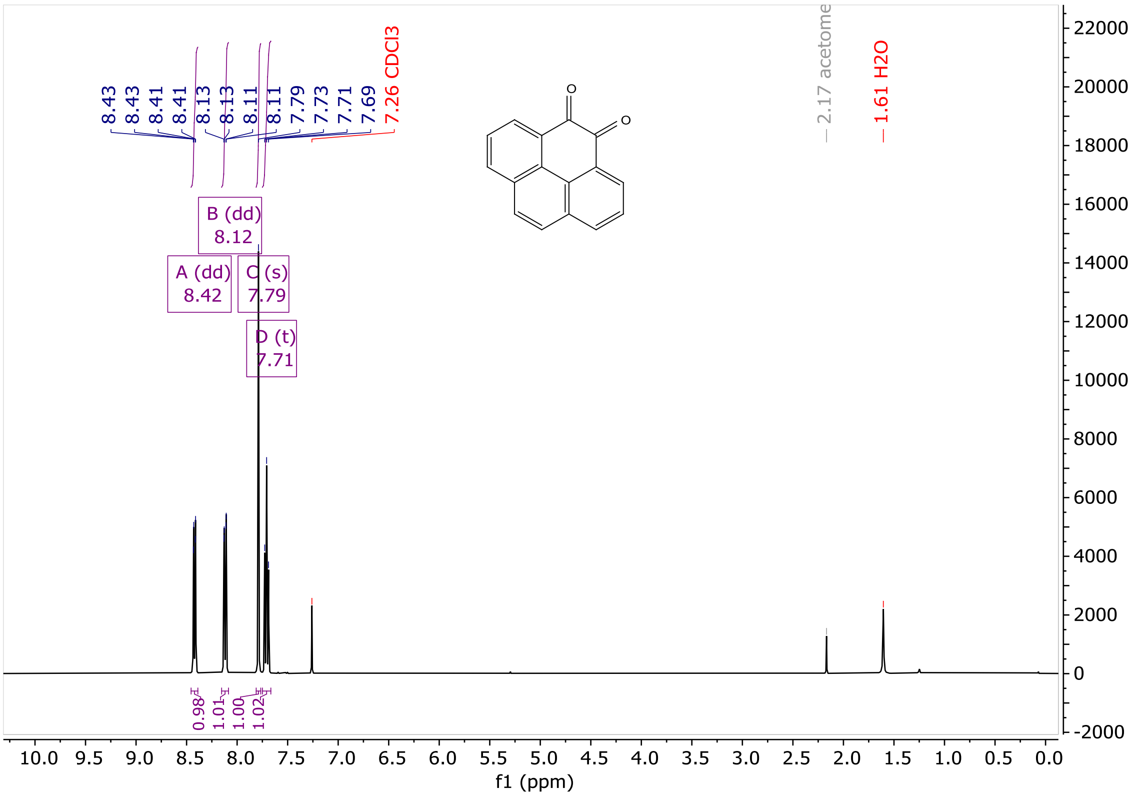

Figure S47: ^{1}H NMR ($CDCl_3$, 400 MHz) spectrum of recovered **PQ**.

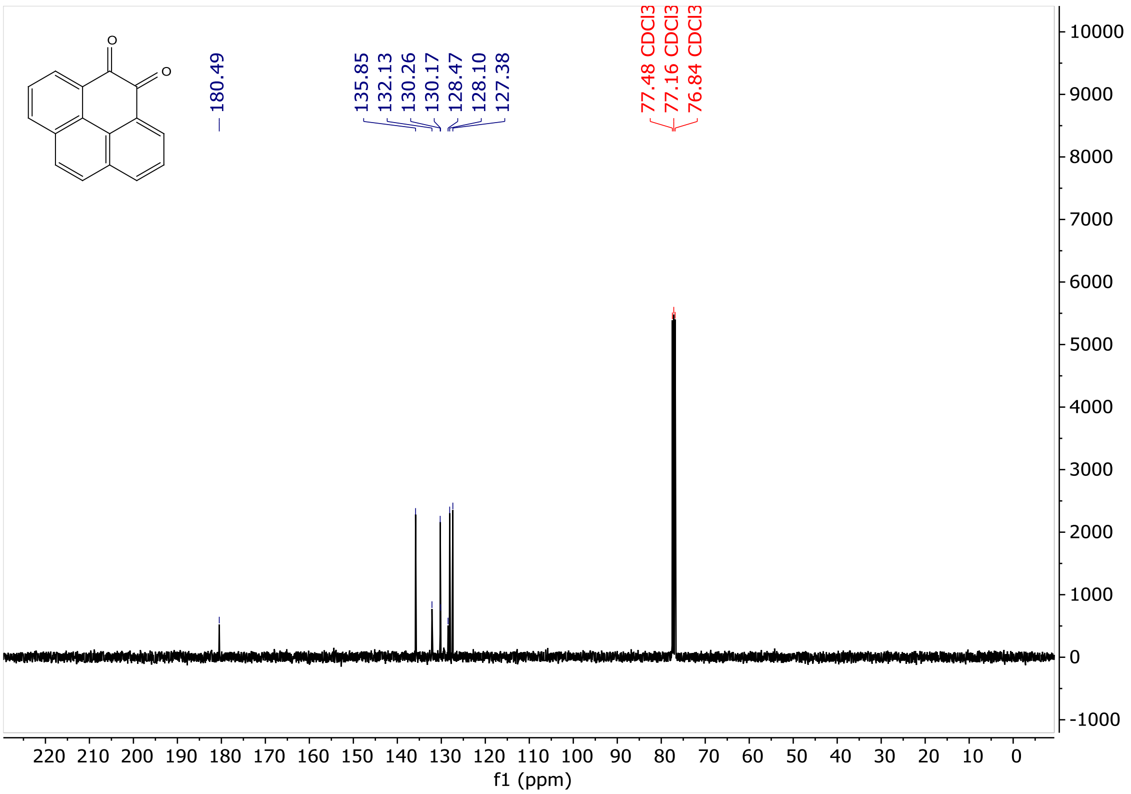

Figure S48: ^{13}C{^{1}H} NMR ($CDCl_3$, 101 MHz) spectrum of recovered **PQ**.

References

1 E. R. R. Young and R. L. Funk, *Journal of Organic Chemistry*, 1998, 63, 9995–9996.
